# Combining Fleetwide AviTeam Aviation Emission Modeling with LCA Perspectives for an Alternative Fuel Impact Assessment

**DOI:** 10.1021/acs.est.3c08592

**Published:** 2024-05-16

**Authors:** Jan Klenner, Marianne T. Lund, Helene Muri, Anders H. Strømman

**Affiliations:** † Industrial Ecology Program, Department of Energy and Process Engineering, Norwegian University of Science and Technology (NTNU), Trondheim 7034, Norway; ‡ Center for International Climate Research (CICERO), Oslo 0349, Norway

**Keywords:** ADS-B, aviation emissions, life cycle assessment, LCA, alternative aviation fuel, SAF, flight fuel consumption model

## Abstract

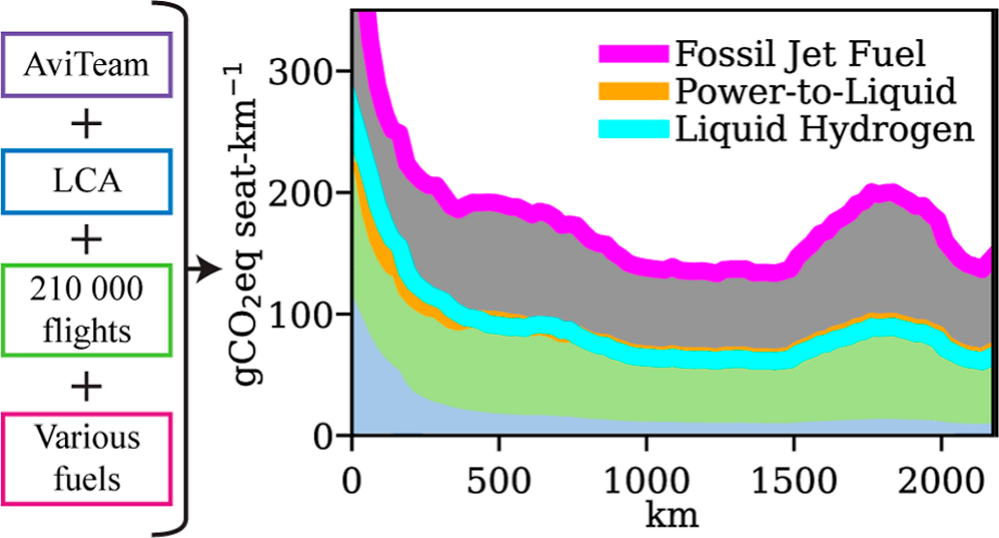

Reducing aviation emissions is important as they contribute to air pollution and climate change. Several alternative aviation fuels that may reduce life cycle emissions have been proposed. Comparative life cycle assessments (LCAs) of fuels are useful for inspecting individual fuels, but systemwide analysis remains difficult. Thus, systematic properties like fleet composition, performance, or emissions and changes to them under alternative fuels can only be partially addressed in LCAs. By integrating the geospatial fuel and emission model, AviTeam, with LCA, we can assess the mitigation potential of a fleetwide use of alternative aviation fuels on 210 000 shorter haul flights. In an optimistic case, liquid hydrogen (LH2) and power-to-liquid fuels, when produced with renewable electricity, may reduce emissions by about 950 GgCO_2_eq when assessed with the GWP100 metric and including non-CO_2_ impacts for all flights considered. Mitigation potentials range from 44% on shorter flights to 56% on longer flights. Alternative aviation fuels’ mitigation potential is limited because of short-lived climate forcings and additional fuel demand to accommodate LH2 fuel. Our results highlight the importance of integrating system models into LCAs and are of value to researchers and decision-makers engaged in climate change mitigation in the aviation and transport sectors.

## Abstract 2

To address literature shortcomings, we combine an aviation fuel burn model with life cycle assessment. We identify limited climatic benefits of alternative fuels, offering insights for aviation’s green transition.

## 1 Introduction

Reducing anthropogenic climate forcings is fundamental to limiting global warming. Aviation contributed about 2.4% to anthropogenic CO_2_^1^ and 1.8% to greenhouse gas (GHG) emissions^2^ in 2018, and the sector’s contribution to global warming is considerably higher due to short-lived climate forcers (SLCFs).^1^ The SLCFs of aviation comprise condensation trails and the subsequent evolution of these to cirrus clouds (collectively called contrail cirrus (CC)), black carbon (BC), organic carbon (OC), nitrogen oxides (NO_*x*_), and sulfur oxides (SO_*x*_) and are collectively estimated to be responsible for about two-thirds of aviation’s global climatic impact (measured in effective radiative forcing).^1,3^

Aviation emission inventories are commonly derived by combining detailed flight information with simplified flight-physics models.^4,5^ Automatic Dependent Surveillance-Broadcast (ADS-B) data is a new, publicly accessible source for geospatially explicit aviation telemetry data available to the community.^6−8^ Models such as the Aviation Transport Emissions Assessment Model (AviTeam) translate these empirical ADS-B data GHG and other emission inventories for aviation.^6,7,9,10^

Those inventories show that aviation emissions have increased steadily over the last decades. The main driver has been the growth in passenger-kilometers at 4.5% per year, which outpaced the average level of efficiency improvements.^1,11^ This trend is projected to continue^12^ after the temporary reduction in air traffic due to the CoViD pandemic.^13^ Consequentially, mitigation measures beyond efficiency improvements, which offer only a limited mitigation potential, are recognized as central to achieving emission reductions in the sector aligned with ambitious climate targets such as a net-zero target by midcentury.^11,14^

One widely considered mitigation strategy is using alternative fuels to replace today’s fossil jet fuels (FJFs), thereby lowering the climatic impact of aviation.^15^ Candidate fuels comprise kerosene synthesized from different carbon feedstocks other than fossil and carbon-free fuels such as hydrogen or ammonia. The Fischer–Tropsch synthesis (FT) is an approved process to create synthesized paraffinic kerosene (SPK) by combining carbon and hydrogen feedstocks.^16^ SPK fuels possess the characteristic of requiring no or minor modification to engine and aircraft design,^17^ which qualifies them for deployment in the near-term option.

Direct air capture (DAC), natural gas, or biogenic feedstocks can provide the required carbon.^18^ DAC could offer particularly low climatic impacts.^18,19^ Amine-based sorbents for DAC are commonly suggested, as early studies indicate a lower environmental impact than alternatives such as calcium carbonate.^20,21^ The use of DAC together with hydrogen produced in alkaline, proton-exchange membrane, or solid oxide electrolyzers is commonly classified under the umbrella term Power-to-Liquid (PtL) fuel.^22−24^ Alkaline electrolysis is the most mature of the three electrolysis technologies.^25^ Studies find, however, that the electrolysis technology is only of secondary importance and that the electricity mix used is a stronger determinant of environmental impacts.^25−27^

In addition, we consider hydrogen fuel, which can be combusted directly or converted to electricity in fuel cells. The limited power–weight ratio is a major disadvantage of current fuel cell technology compared to direct combustion.^28,29^ Hence, we focus on direct combustion and liquid hydrogen (LH2) as storage technology, which has received more attention (e.g., refs^30−33^) than compressed gaseous hydrogen for its higher volumetric density. Yet, LH2’s volumetric energy density is 80% lower than kerosene’s, which would imply changes to aircraft design and operation, making LH2’s practical deployment appear a more distant possibility.^11,33,34^ Several system studies have proposed and assessed different mitigation scenarios that combine alternative aviation fuels, efficiency increases, and other measures.^33,35−39^ For these scenarios, sometimes, a single value for the climatic impact of alternative fuel production (FP) is used, commonly derived with life cycle assessment (LCA),^33,35^ and sometimes, net zero life cycle CO_2_ emissions are assumed.^36,37^

Emission metrics are often used in LCAs to express impacts of SLCFs and other GHGs relative to CO_2_. The derivation of emission metrics is well established and documented in the existing literature (e.g., refs^40−44^). A very common metric in the LCA community, despite criticism,^45,46^ is the global warming potential (GWP) for a 100 year horizon (GWP100). Metrics imply a weighting between SLCFs and long-lived climate forcers which usually depends on the time scale chosen.^46^ Thus, when using metrics, presenting a set of different metrics can be beneficial.^47^

Systemwide studies integrating an LCA perspective of alternative aviation fuels are rare. Most LCA studies rely, if at all, on external sources for fleet properties like fuel burn and emissions (e.g., refs^19,23,31,48,49^). Therefore, they tend to compare fuels on their energy values, thereby implicitly assuming that one MJ of FJF is equivalent to one MJ of LH2 fuel.^19,23,31,48,49^ This assumption of equality, however, can be questioned as LH2 fuel may be lighter but also less dense and thus alter the energy demand of the aircraft fleet for providing the same service (passengers transported a certain distance). Miller et al.^30^ adjusted LH2 fuel demands with a fleetwide factor, however, without considering systemic variability such as extra fuel volume needs varying with flight distance. Previous well-to-wake (comprising FP and combustion) LCA studies report mitigation potentials in the GWP100 metric of 46–72% for PtL^19,23,48^ and 40–99% for LH2 aviation fuels^30,31,49,50^ when produced with renewable electricity. However, some of these studies do not quantify all the SLCFs^19,31,49^ of aviation, and others^23,48^ are not consistent with the International Panel on Climate Change (IPCC) Sixth Assessment Report climate functions for metrics calculation,^51^ thus potentially underestimating the impacts of alternative fuels.

This work presents the results of a systemwide comparison of alternative aviation fuels for shorter haul flights where a fuel burn model and the LCA framework are combined. With high-resolution modeling of 210 000 flights using the AviTeam framework,^6,9^ we can represent fleetwide fuel burn and emissions of varying fuels. Moving beyond analyses of a few aircraft–distance pairs, we can (i) maintain a detailed FP modeling, (ii) endogenize the implications of a fuel switch on the entire fleet’s energy demand for shorter haul flights, (iii) capture operational variability in the mitigation potential of alternative fuels, and (iv) integrate highly detailed aviation emission estimates and life cycle thinking to discuss the balance of short-lived and long-lived climate forcings and the mitigation potential of alternative aviation fuels.

## 2 Methods

We assess the mitigation potential of alternative aviation fuels for shorter haul flights. For this purpose, we combine the Aviation Transport Emission Assessment Model (AviTeam) with LCA to create high-fidelity well-to-wake inventories. Our flight data comprises 210 000 domestic flights from Norway in 2019. The model we use to derive fuel consumption and emissions and the data set have previously been described and benchmarked in Klenner et al.^6^

In AviTeam, the energy requirement per flight is calculated with the Eurocontrol Base of Aircraft Data 3 Version 15 (BADA 3) aircraft performance model.^52^ Compared to the AviTeam version of Klenner et al.,^6^ we reduce complexity in modeling LH2 fuel by grouping aircraft into 11 clusters representing different engine types, aircraft sizes, and ages (Supporting Information Table S1). We choose a representative aircraft for each cluster and assume that those representative aircraft perform all flights of their cluster, introducing uncertainty in flight fuel consumption of about 20%. In the LH2 case, we choose a larger representative aircraft to perform the flight to ensure that enough space is available for the seats and the hydrogen trip fuel. We assume that the larger representative aircraft is equipped with the same number of seats as the original representative, such that the difference in fuselage volume between the original and larger aircraft can be dedicated to the hydrogen tank system. We calculate the minimum fuel tank size needed for hydrogen based on the actual energy consumption, which results in different size increments for different representative aircraft. We further assume an identical engine efficiency for FJF, SPK, and LH2 fueled engines (c.f., ref (53)). The fuel weight is explicitly considered in the fuel burn calculation and in the aircraft mass updates along the flight path. Freight transport is not considered separately and instead translated to passenger flights assuming a standard seating, which affects 2.3% dedicated freight flights and passenger flights that carry extra freight. The aircraft clustering, aircraft mass calculation, and further additions to AviTeam are described in detail in Supporting Information Section S.1.

Emissions from fuel combustion are modeled linearly (CO_2_, H_2_O, SO_*x*_, and OC) and nonlinearly (NO_*x*_, HC, CO, and BC) to fuel burn. Emission indices for FJF and alternative fuels used in this study are provided in Table 1. NO_*x*_, HC, and CO emissions are calculated using the Boeing Fuel Flow Method 2, introduced and described in detail by Dubois et al.^54^ This method utilizes the emission measurements provided in the ICAO and FOCA engine emission databases^55,56^ and adjusts emission indices (in kg emission per kg fuel burn) measured at ground-level conditions to the atmospheric ambient conditions (pressure, humidity, and temperature). This modeling approach has been described in the original description of the AviTeam model.^6^ BC emissions, expressed on a mass basis, are modeled similarly to the other nonlinear emissions. The approach is described in Supporting Information Section S.1 and was introduced by Quadros et al.^7^

**Table 1:** Emission Indices for Different Fuels^a^

species	FJF [g MJ-fuel–1]		SPK [%]		LH2 [%]	
CO_2_	73.32	H	100	H	0	H
H_2_O	28.56	H	100	H	260	H
SO_*x*_	2.79 × 10^–2^	H	0	H	0	H
OC	6.96 × 10^–6^	H	25 (10, 50)	L	0 (0, 50)	L
BC_m_	var	M	25 (10, 50)	L	0	H
NO_*x*_	var	H	100	M	35 (10, 110)	VL
HC	var	H	90 (80, 100)	M	0	H
CO	var	H	90 (80, 100)	M	0	H
LHV (MJ kg^–1^)	43		43		120	

a Emissions in g MJ-fuel^–1^ for FJF. Emissions for SPK and LH2 are expressed relative to FJF emissions, and those expressed in %. “var” indicate variable, nonlinear emission modeling in AviTeam. LHV: lower heating value. BC_m_: BC mass. Ranges used in the sensitivity study are provided in parentheses. Sources for LH2 emissions:.^17,68−70^ The contribution of lubricating oils to total OC emissions is uncertain, and a reduction of 100% (50–100% interval) is assumed. Sources and further information for SPK in Supporting Information Section S.2. Letters indicate relative confidence in the emission index: High (H), medium (M), low (L), and very low (VL).

We express the emissions of alternative fuels normalized with FJF emissions per MJ-fuel. The change in emissions under SPK fuel is modeled based on a literature review summarized in Supporting Information Section S.2. A constant relationship between alternative fuel emissions and FJF emissions is assumed in the absence of flight stage or engine-specific emission values.

In addition to direct emissions, we include an indication of the CC impacts, which depend on the composition of aviation emissions and the ambient atmosphere. The driving factors are the water content and aerosol emission number and diameter.^57,58^ SPK has a lower aromatic and naphthalene content and higher paraffinic content^59−62^ and is associated with a reduced number of particle emissions.^62−65^ This implies an expected reduction in contrail lifetime and CC’ radiative forcing.^66,67^ For CC impacts of FJF, global average values normalized per kg fuel from Lee et al.^1^ are used as the explicit estimation of historic contrail formation, and forcings are beyond current capabilities of AviTeam. In agreement with Dray et al.,^33^ we model SPK fuels’ CC impacts as 58% of FJF impacts.

Studies of CC impacts and NO_*x*_ emissions of hydrogen-fueled aircraft are more sparse.^17,68−70^ Collectively, the literature suggests that in the case of hydrogen combustion, the average warming impact of CC per MJ-fuel will be reduced due to a reduced number of ice nuclei, analogously to FT fuels.^33,70,71^ Further, the literature suggests that the minimal, stable flame temperature achievable with hydrogen is lower than that of conventional kerosene combustors.^64,68,72^ This may allow for a significant reduction in thermally produced and overall NO_*x*_ emissions.^72,73^ We use a LH2 CC impact as 85%^33^ and NO_*x*_ emissions as 35% of FJF’s per MJ-fuel. Aerosol–cloud interactions are not included for any fuels because of large related uncertainties.^1^

### 2.1 Quantification of Aviation Emission Impacts

The climate impacts of aviation emissions are estimated in terms of CO_2_-equivalent emissions using emission metrics in line with traditional LCA methodology. An updated set of emission metrics for aviation emissions is calculated for the current study using the current best estimates of global mean radiative forcing of aviation emissions (Data set 1). Specifically, we calculate the GWP and global temperature change potential (GTP) for aviation H_2_O, SO_*x*_, NO_*x*_, CO, BC, OC, and CC, for a 20, 50, and 100 year horizon. We refer the reader for methodological details on metrics calculations to Lashof et al.,^40^ Shine et al.,^41^ Fuglestvedt et al.,^42^ Aamaas et al.,^43^ and Myhre et al.^44^ Our updated metrics use the best estimate of effective radiative forcing for aviation non-CO_2_ effects from Lee et al.^1^ (with the exception of OC, which was not included in Lee et al.^1^ and where we use the global mean RF value from Lund et al.^3^ instead) and are broadly similar to the values reported there, but the calculations include three updates: (i) we use the impulse response function for temperature response that is consistent with the IPCC AR6^51^ instead of the one from Boucher et al.^74^ that is commonly used in previous calculations, (ii) we include an estimate of the carbon-climate feedback in the SLCF emission metrics following the approach by Gasser et al.,^75^ and (iii) we update the GHG emission metrics to year 2019 atmospheric concentration levels using the equations from Etminan et al.^76^ These assumptions explain differences in the GWP values compared to Lee et al.^1^ We apply the aviation-specific metrics to emissions in the operational phase and the GWP/GTP metrics from Myhre et al.^44^ to the remaining life cycle GHG and SLCF emissions.

We note that our analysis builds on the assumption of a fuel switch for shorter haul flights equivalent to domestic aviation. The impacts of SLCF emissions can depend strongly on the location of emissions and the background concentrations. Hence, using emission metrics derived from the global fleet may be an over- or underestimate of the climatic impacts of regional flights. Geographically specific metrics are not readily available and could complicate a comparison with other works; however, alternative approaches for larger scale scenarios are discussed later.

Further, the metric of cumulative energy demand (CED) is used to measure the energy requirements for different aviation fuels. The metric measures the higher heating value of energy harvested from renewable and nonrenewable sources.^77^ We also provide results for the ReCiPe 2016 Hierarchist Midpoint indicators^78^ in Supporting Information Table S3.

### 2.2 Fuel Production

To complete the well-to-wake perspective, we explicitly model the production of the fuels assessed (Supporting Information Figures S12–S19). As no flight-specific passenger numbers are available, we use a functional unit in the LCA of available seat-km of commercial passenger aircraft.

We use an attributional LCA approach where infrastructure construction and end-of-life treatment are included in the inventories. The background system is modeled with the ecoinvent 3.8 (cutoff) database.^79^ We separate the foreground into the phases of resource extraction, FP, transport and storage, and the operation modeled as aforementioned. Resource extraction, energy production, FP, transport, and refueling are assumed to be located in Norway; other processes are modeled with their global market mixes. The assumed production in Norway affects the electricity mix for alternative fuels, which is a key factor (Supporting Information Figure S6), but has limited influence on (fossil) resource extraction and fuel transport (Supporting Information Figure S5).

Natural gas, crude oil extraction, and oil refinement are modeled explicitly using publicly available emission inventories from the Norwegian Environment Agency, averaged over the years 2017–2019^80^ (Supporting Information Tables S5–S11). A sample of five platforms is chosen. These emission inventories are complemented with material flows from Wernet et al.^79^ We calculate the transport distances for natural gas (pipelines) and crude oil (pipelines and tankers) to the only refinery in Norway, located in Mongstad, based on geospatial information on pipelines^81^ (Supporting Information Tables S9 and S12). SPK production is modeled with FT synthesis. For all extraction and refinery processes, energy-content-based allocation is chosen. Electrolysis of hydrogen, hydrogen liquefaction, and DAC are assumed powered by onshore wind power, which represents low-carbon energy sources (GWP100:13 gCO_2_eq kWh^–1^).

We assume that fuel is produced case dependently either at centralized locations or decentralized at the airport locations. In the case of centralized production, we assume the location of Norway’s only operational refinery. Transport from there to the airport is modeled as road transport, and transport distances are calculated using OpenStreetMap;^82^ in the case of alternative fuels, transport is fueled with LH2 (Supporting Information Table S16). The impacts of storage infrastructure before the aircraft fueling are neglected. Losses for transport and storage are assumed to be zero except in the case of LH2. There, boil-off losses of LH2 are taken into account with a fuel loss of 0.5% for road transport and a loss equivalent to three storage days with 0.1% boil-off per day.^83^ We include the construction and operation of airport infrastructure. Their impacts are retrieved from ref^79^ and converted to impacts per seat-km of departing flights. For this, we use a lifetime of 100 years, an annual passenger equivalent of 29.2 million passengers,^84^ and the average load factor for Norway in 2019 of 69.23%.^85,86^ Aircraft construction and end of life are also considered by scaling values from Wernet et al.^79^ with the aircraft’ empty weights.

In the main article, we compare FJF (used as the reference for mitigation potentials in this work) with one representative LH2 and SPK fuel produced via low-impact pathways. As a representative pathway of LH2 fuels, we choose LH2 fuel from hydrogen produced with alkaline electrolysis, as it is the most mature electrolysis technology, and using electricity from wind power (LH2-W). To represent SPK fuels, we use PtL fuel from DAC with an amine-based sorbent combined with hydrogen from alkaline electrolysis using electricity from wind power to guarantee comparability (PtL-W). Overall, the selected FP pathways for alternative fuels can be seen as an optimistic estimation as they use low-impact electricity from wind power, and technology parameters demonstrated only at smaller scales are used for large-scale systems. In the Supporting Information, we present results for other hydrogen and carbon feedstocks, namely, the gas-to-liquid process, autothermal reforming, steam–methane reforming, DAC with calcium carbonate sorbent, and solid–oxide electrolysis (Supporting Information Figures S8 and S9).

## 3 Results

To assess the mitigation potential of alternative fuels, we apply the AviTeam framework combined with life cycle modeling to shorter haul flights with distances from 40 to 2 200 km. First, we present a fleetwide results including variations with flight distance. Then, we inspect at different time scales to illustrate the trade-offs between SLCF and CO_2_ impacts. Last, we present a more detailed view of the CED.

### 3.1 Climatic Impacts and Mitigation Potentials Vary with Flight Distance

**Figure 1:**
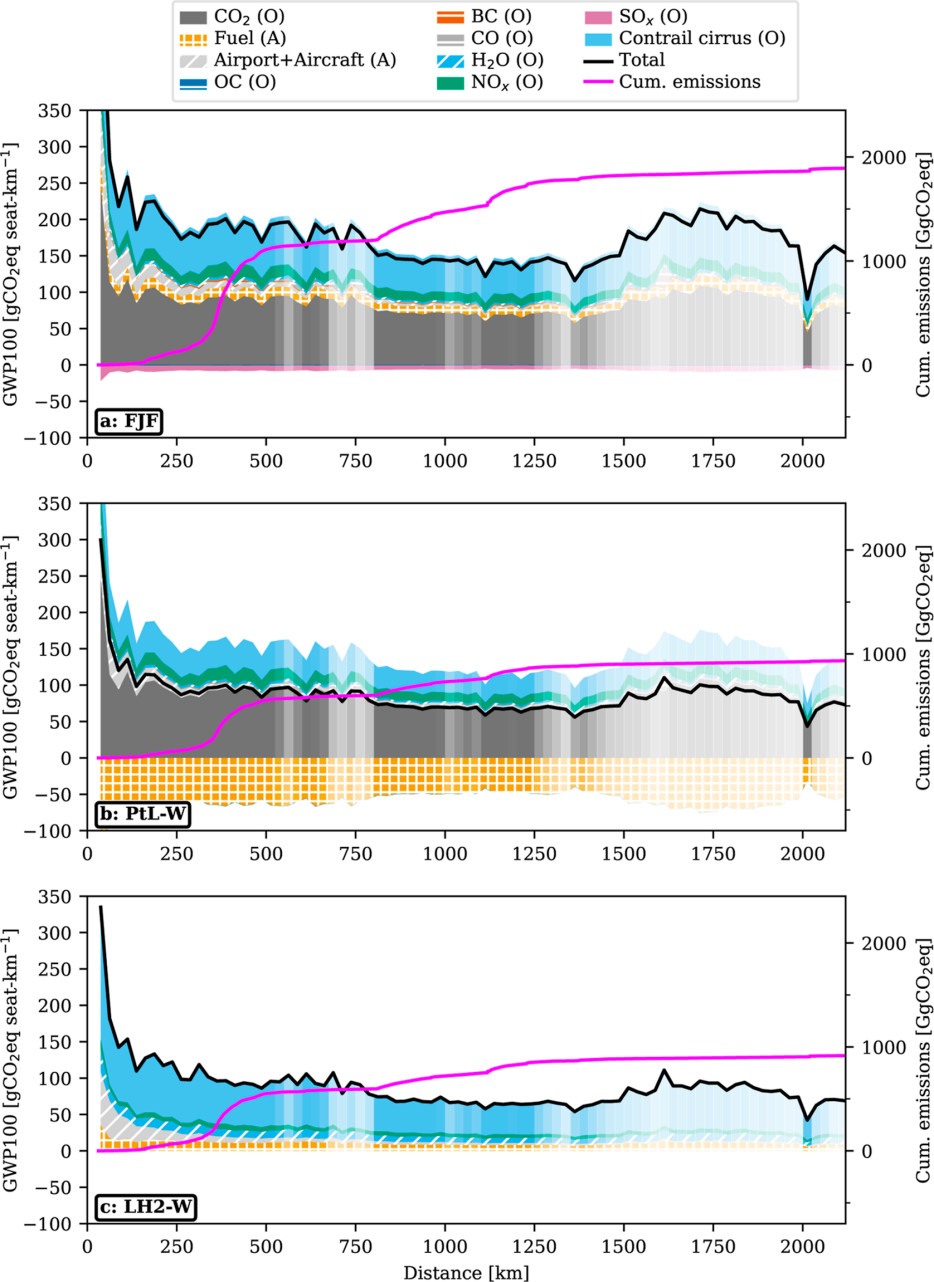
CO_2_-equivalent emissions of FJF, PtL-W, and LH2-W flights by distance. a: Fossil jet fuel (FJF); b: power-to-liquid fuel with amine-based DAC and alkaline electrolysis (PtL-W); c: LH2 from wind power and alkaline electrolysis (LH2-W). First axis (left side): mean CO_2_-equivalent emissions per seat-km of 210 000 flights. Flight distances in km (*x*-axis) and CO_2_-equivalent emissions in GWP100 in gCO_2_eq seat-km^–1^ (*y*-axis). Operational impacts are shown disaggregated into the mean contribution of individual emission species (O). Fuel, aircraft, and airport emissions are shown in aggregated form (A). Negative, i.e., cooling contributions are stacked below the zero line. The total (black line) describes the sum of all individual components. Color intensity is increasing with the number of flights per bin. Values are calculated for 25 km bins. Second axis (right side): fleetwide cumulative CO_2_-equivalent emissions (in GgCO_2_eq) for the 210 000 flights used in this analysis as a red line.

Fleetwide cumulative emissions using FJF flights sum to 1890 GgCO_2_eq using the GWP100 metric, PtL-W emissions to 940 GgCO_2_eq, and LH2-W emissions to 920 GgCO_2_eq. Very short FJF flights of less than 200 km have an average total impact (black line in Figure 1) of 228 gCO_2_eq seat-km^–1^ (PtL: 119 and LH2:128). Meanwhile, the longest FJF flights considered (>2 000 km) average at 100 gCO_2_eq seat-km^–1^ (PtL-W: 48 and LH2-W: 46), thus showing lower impacts by more than a factor of 2. The implied mitigation potential of PtL-W fuel on the shortest flights (<200 km) is 48%, which is slightly larger than LH2-W fuels’ 44% (Supporting Information Figure S7). On longer flights, average potentials increase to 52% (PtL-W) and 54% (LH2-W). The increase in the emission for distances between 1 300 and 2 000 km coincides with a low number of flights and hence a larger uncertainty (Figure 3). Thus, we classify the rapid changes as an artifact of the data set in use. The impact curve for flights of around 2 000 km continues the general trend of distances ≤ 1 300 km, as the number of observations is again larger.

Several factors explain the lower GWP per seat-km of longer flights. First, the energy consumption per seat-km is lower on longer flights with a reduced contribution of the energy-intensive climb stage (compare Figure 3b). Second, the contribution of airport infrastructure per seat-km is lower for longer flights due to an attribution per seat regardless of the flight distance. Third, the average number of seats per aircraft increases with distance in our data set (Supporting Information Figures S10 and S11). Energy economies of scale are present and reduce energy consumption and hence the climatic impact per seat-km. The slightly larger reduction of impacts in relative terms with larger distances in the LH2-W case compared to PtL-W and FJF is explained by a larger contribution of operational emissions on longer flights, mainly CO_2_ and CC, and changes in CED, that show how the additional energy demand for LH2 flights is particularly large in our data for flights in the shortest segments, presented later. Fourth, the composition of the fleet changes. In our data, LH2-W performs better for jet aircraft than turboprop and piston aircraft (Supporting Information Figure S7), the latter having a larger share on shorter flights.

### 3.2 Climatic Impacts of Alternative Aviation Fuels Vary with the Time Horizon and Metric

**Figure 2:**
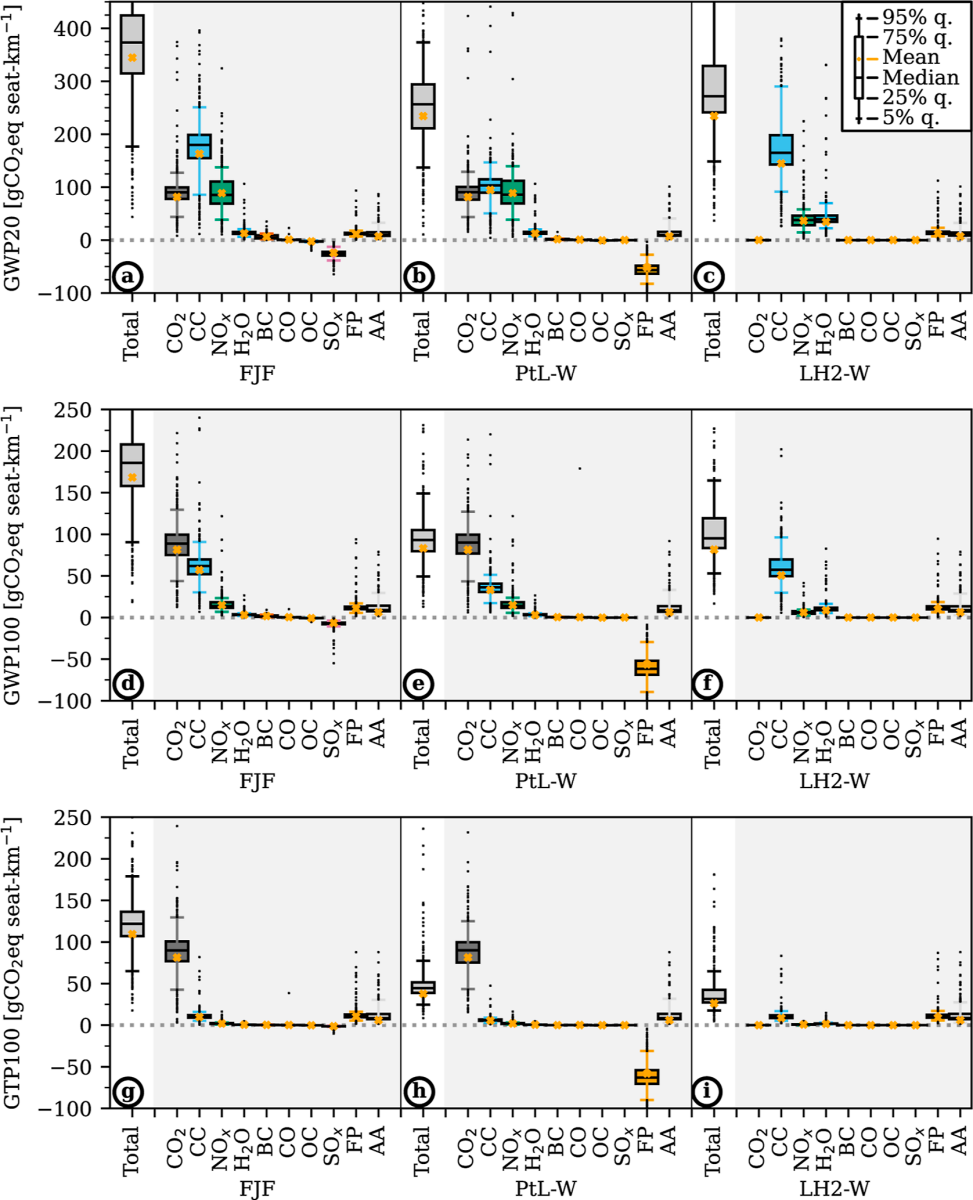
CO_2_-equivalent emissions per seat-km^–1^ of selected fuels as totals and by individual contributions using different metrics. *y*-axis in gCO_2_eq seat-km^–1^. GWP20 results for (a) FJF: fossil jet fuel (kerosene), (b) PtL-W: power-to-liquid fuel with amine-based DAC and alkaline electrolysis using electricity from wind power, (c) LH2-W: LH2 using electricity from wind power and alkaline electrolysis. GWP100 results in (d)–(f), GTP100 results in (g)–(i). Total CO_2_-equivalent emissions are further broken down into the contribution of individual operational emissions [CO_2_, CC, NO_*x*_ (H_2_O, BC, carbon monoxide (CO), OC, and SO_*x*_], FP, and aircraft production and airport operation (AA). FP impacts of PtL fuel include CO_2_ adsorbed in DAC. Values for all flights in boxplots with weighted mean (orange marker), median (black horizontal lines), 25 and 75% quantiles as box edges, and 5 and 95% quantiles as whisker positions, and other values as small, black dots.

The assessed mitigation potential of alternative fuels varies not only with the flight distance but also with the choice of emission metric and time horizon over which the climate impact is evaluated as the balance of short-lived and long-lived climate forcings changes over time (Figure 2). SLCF impacts dominate the GWP, 20 year time horizon (GWP20). The mean GWP20 of FJF flights is 345 gCO_2_eq seat-km^–1^. GHGs (CO_2_ and H_2_O) and SLCFs (CC, SO_*x*_, NO_*x*_, CO, BC, and OC) of the operational phase contribute 94 and 232 gCO_2_eq seat-km^–1^, respectively. We calculate a mean GWP20 for LH2-W and PtL-W fuel of 234 gCO_2_eq seat-km^–1^, hence 32% lower values compared to FJF. The reduced impact of alternative fuels is explained by a reduction in SLCFs and a net zero contribution of operational CO_2_ as CO_2_ is captured from the atmosphere during SPK FP, and avoided altogether in the LH2 case.

We observe lower GWP100 scores for fuels due to the reduced importance of SLCFs on longer time scales, particularly those of CC and NO_*x*_, responsible for the bulk of impacts in GWP20. Vice versa, the relative contribution of long-lived CO_2_ is larger. This is an inherent feature of the GWP, as SLCF impulses decay faster and thus contribute less than long-lived CO_2_ on longer time scales. FJF impacts average 168 gCO_2_eq seat-km^–1^, with CO_2_ being the largest single contributor. PtL-W’s impacts average 83 gCO_2_eq seat-km^–1^ and LH2-W’s 82 gCO_2_eq seat-km^–1^, thus offering a mitigation potential of 51 and 52%, respectively.

The contribution of SLCFs further decreases in the GTP, 100 year horizon (GTP100) metric, which describes the CO_2_-equivalent emissions, leading to the same temperature change in the end year, thus giving more weight to long-term impacts. In the case of FJF, 81 of 110 gCO_2_eq seat-km^–1^ are attributed to CO_2_. PtL and LH2 cause a mean impact of 38 gCO_2_eq seat-km^–1^ (−65%) and 26 gCO_2_eq seat-km^–1^ (−76%), respectively, thus showing the largest mitigation potential across all the assessed metrics. The results suggest that mean LH2-W impacts are slightly below those of PtL-W caused by lower NO_*x*_ and FP impacts, which outweigh increased CC impacts compared to PtL fuel. The contribution of airport and aircraft infrastructure to average impacts is of subordinate nature in all metrics.

The spread in impacts (Figure 2) is caused by the aircraft and engine types and other operational factors such as higher travel speeds or operational inefficiencies. The boxplots summarize the large variability in impacts (e.g., interquartile range for FJF extends from 158 to 208 gCO_2_eq seat-km^–1^, PtL-W: 80–105, and LH2-W: 83–119) that could inform studies that are not fleetwide and hence simplify the fleet composition and flight data set.

LH2-W and PtL-W perform comparably across all metrics and 30–70% better than FJF. These results are subject to significant uncertainties as the technological readiness of PtL and LH2 is low, and CC impacts uncertain. However, the results highlight that the mitigation potential of alternative aviation fuels is limited in the near-term perspective and growing when considering longer time scales. In the long term, LH2-W shows a slightly larger potential than PtL-W due to the limited importance of SLCFs and lower FP impacts per seat-km.

### 3.3 CED under Varying Flight Lengths

**Figure 3:**
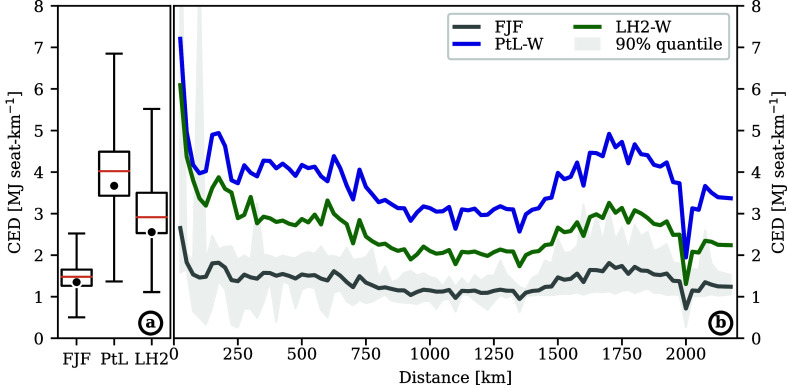
CED of alternative fuels by flight distance. FJF: fossil jet fuel (kerosene), PtL: power-to-liquid fuel with amine-based DAC and alkaline electrolysis using electricity from wind power (PtL-W), and LH2: liquid hydrogen using electricity from wind power and alkaline electrolysis (LH2-W). (a) Boxplot of CED of all flights in MJ seat-km^–1^. Equal weighting across the entire data set. The plot shows the medians (orange line), means (black dots), interquartile ranges (box edges), and 5 and 95% quantiles (whiskers). (b) Mean CED in MJ seat-km^–1^ by flight distance [km] for fossil jet fuel (gray), PtL (blue), and LH2 (green). 5–95% quantile range of FJF as shaded gray area. PtL and LH2 quantile ranges follow a similar, offset distribution. Means and ranges are calculated for 25 km bins.

**Figure 4:**
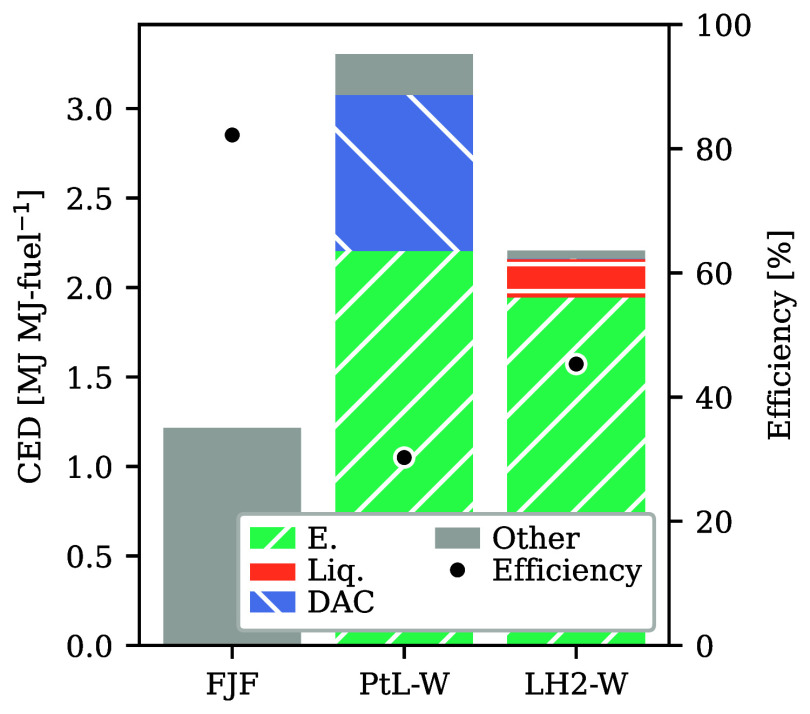
CED of aviation FP. CED in MJ to produce one MJ aviation fuel (bars, left axis) and energy efficiency in percent (black dots, right axis) as applied to the data set. FJF: fossil jet fuel (kerosene), PtL-W: power-to-liquid fuel with amine-based DAC and alkaline electrolysis using electricity from wind power, LH2-W: liquid hydrogen using electricity from wind power and alkaline electrolysis. Colors encode the direct energy demand of electrolysis (E., green), hydrogen liquefaction (Liq., orange), direct air capture (DAC, blue), and others (gray).

Differences in the climatic impact per seat-km are related to the CED (Figure 3). The total CED is the product of fuel demand per seat-km [MJ-fuel seat-km^–1^] and CED of FP [MJ MJ-fuel^–1^]. We find that the mean CED increases significantly from FJF to LH2-W and PtL-W systems and observe a large variability between flights (Figure 3a). In Figure 3b, we partially unravel this variability by showing the mean CED per seat-km of flights grouped into 25 km distance bins. Fuels are shown by separate lines as there are nonlinear variations between LH2 and carbon-based PtL and FJF. Further, variability within each distance group is showcased by the gray-shaded 5% to the 95% quantiles range of CED for FJF. On the shortest flights, the CED of PtL-W and LH2-W flights is almost identical despite the higher energy demand of PtL-W in FP (Figure 4). This is explained by a higher fuel demand by LH2 flights in general and on shorter flights in particular, which partially offsets a lower energy demand during the LH2-W production. Across all flight distances, LH2 flights consume 1.15 MJ-fuel seat-km^–1^ and thereby 4% more than FJF and PtL flights (1.11 MJ-fuel seat-km^–1^). The higher fuel demand in the LH2 case is explained by the need to accommodate larger fuel tanks and, thus, the use of larger aircraft.

The CED in the FP of FJF, PtL-W, and LH2-W is calculated as 1.22, 3.3, and 2.2 MJ MJ-fuel^–1^, respectively (Figure 4). These energy demand values translate to respective total energy efficiencies (as the ratio of final fuel energy content to energy harvested from renewable and nonrenewable sources) in the FP of 82% (FJF), 30% (PtL-A), and 45% (LH2-WA), showcasing inefficiencies, particularly in electrolysis, in the production of these alternative aviation fuels compared to FJF. However, FJF uses fossil energy resources, while PtL-W and LH2-W are produced with renewables. The PtL-W production requires the most energy in the form of electricity in the alkaline electrolysis and DAC. In the case of LH2-W, the main processes are alkaline electrolysis and liquefaction.

Key sensitivities and uncertainties are quantified for the GWP100 case to complement the previous results (Supporting Information Figure S6). The analysis highlights the importance of using low-impact energy resources in the FP stage, as alternative electricity mixes with a much higher impact per kWh than the assumed 13 gCO_2_eq kWh^–1^ lead to scenarios with FJF performing best. The assumed uncertainty in emission indices, particularly CC, and the uncertainty related to GWP estimates of aviation emissions may reverse the order of PtL-W and LH2-W, but a scenario where FJF performs better appears unlikely. Furthermore, alternative FP pathways provide mitigation potentials relative to FJF for PtL in the range of 73–93 gCO_2_eq seat-km^–1^ and for LH2 47–86 gCO_2_eq seat-km^–1^ (Supporting Information Figure S8).

## 4 Discussion

In our work, we combine the AviTeam fuel consumption model and LCA to assess the climatic impacts of fleetwide use of alternative aviation fuels leveraging a data set of 210 000 shorter haul (domestic) flights. With our method, we can represent inherent variability in climatic impacts of different flights and take into account fuel properties such as volume and weight on the climatic impact and mitigation potential. In our analysis, a fleetwide deployment of PtL-W and LH2-W fuels offers mitigation potentials compared to FJF fuel flights’ of cumulatively 960 GgCO_2_eq and 980 GgCO_2_eq, respectively. The mitigation potentials of PtL-W and LH2-W systems are 48 and 44%, respectively, on the shortest flights (≤200 km) compared to FJF fuel flights’ 228 gCO_2_eq seat-km^–1^. On longer flights, the mitigation potentials increase to 52% (PtL-W) and 54% (LH2-W) compared to FJF flights’ 104 gCO_2_eq seat-km^–1^.

Our results may inform fleetwide research and policy when comparing alternative aviation fuels as they consider how climatic impacts and mitigation potentials vary with flight distance, fleet composition, and other parameters. This highlights the benefits of considering these aspects in policy-making and when comparing research results from different sources. Results also show that using LH2 fuel may be less advantageous in specific fleet segments, such as turboprop and piston aircraft flying less than 1000 km (Supporting Information Figure S7). A fuel demand (and thus climatic impacts) per seat-km decreasing with flight distance (for flights shorter than 5 000 km) and depending on the aircraft type aligns with findings from Proesmans et al.,^32^ Cox et al.,^84^ and Graver et al.^87^ As the aircraft in our data set may have a lower average age than in other regions, our results may underestimate the current fuel efficiency and mitigation potential of fuels, and results may be closer to mitigation potentials for future fleets in other regions. We show that results depend to a certain degree on the system parameters; hence, ideally, similar analyses are applied if the scope is changed.

The LH2-W fuel’s mitigation potential of 45–55% (GWP100) is substantially lower than in some LCA studies using renewable electricity^31,50^ because our results include the SLCF of aviation based on the latest literature. Our results are closer to 40–70% reduced life cycle impacts for LH2 fuels from renewable electricity identified by Miller et al.^30^ and Dray et al.^33^ FP impacts in our study confirm previous results for FJF,^79^ electrolysis,^88,89^ and PtL production from renewable electricity sources.^18,23,48,90^

As of today, results for LH2 and SPK are hypothetical as neither LH2 fuels nor SPK fuels have reached market readiness, given a low technological readiness, particularly for LH2 systems, and a significant cost premium of both alternative fuels compared to FJF.^11,32,33,91^ This is a relevant source of uncertainty. Hence, current results are indicative, without a clear ranking between LH2 and SPK. Future evaluations should use updated data for FP pathways and emission indices to confirm or improve current estimates.

We identify varying fleetwide energy demands for different fuels, as our method allows us to quantify the additional energy needed to accommodate lower density fuels such as hydrogen. This is another factor in explaining the lower mitigation potential of LH2 fuel compared to some comparative LCAs, which do not take into account system implications. This study’s LH2 energy demand increase of 4% is smaller than a 8–14% increase identified by Proesmans et al.^32^ for reshaped regional aircraft. While we do not provide quantification of longer international flights, results from Proesmans et al.^32^ suggest that a fuel penalty will be larger on longer hydrogen flights. The potentially larger energy demand for fleets powered by low-density fuels ought to be considered when quantifying environmental impacts and when defining guidelines and policies for alternative aviation fuels.

SLCFs dominate in the short term, and hence, the LH2 and PtL fuel’s mitigation potentials are limited as they offer limited reductions of SLCF emissions. The exclusion of aerosol–cloud interactions and the uncertainty related to CC impacts of alternative aviation fuels add uncertainty to the results, particularly for shorter time scales. We build our analysis of CC on the current literature regarding impacts at the global scale, but there are several sources of uncertainty, namely, (i) the current contribution of CC,^92,93^ (ii) the reduction of cloud condensation nuclei from alternative fuels (Supporting Information Figure S4), (iii) how this will alter the radiative forcing from CC relative to CO_2_ emissions,^94,95^ and (iv) differences between world regions.^93^ Last, CC formation is more likely at specific altitudes,^96^ which we have approximated in the sensitivity analysis (Supporting Information Figure S6) and found to be of subordinate relevance in our case. Regardless, on longer time scales, CO_2_ impacts become more important, and alternative fuels will likely hold a larger mitigation potential than on short time scales.

The large share of SLCFs complicates the metrics-based quantification of mitigation potentials, most notably how to weigh SLCF against CO_2_ impacts.^43,45−47^ Larger SLCFs can further imply a trade-off between mitigation in the short term (e.g., non-GHG impacts such as CC) and mitigation in the long term (CO_2_), for instance, in contrail avoidance.^94^ Also, SLCFs have a strong local component,^3^ encouraging a spatial disaggregation of inventories and metrics in future LCA of aviation fuels as well as further research on the impacts of alternative aviation fuels on atmospheric chemistry and radiation. More complex assessments of aviation’s SLCF and total emissions, such as demonstrated by Dray et al.,^33^ Grewe et al.,^35^ Bergero et al.,^36^ Klöwer et al.,^37^ and Brazzola et al.,^38^ may be preferable when available at a reasonable cost. Regardless, the results of our and similar studies can play an important role by providing detailed information on fuel consumption, emissions, certain modeling aspects, and life cycle information on fuels to other studies and scenarios.

Beyond the drawback of limited technological readiness,^25,30^ alternative aviation fuels also imply a large energy demand for their production. In our analysis, the demand by PtL and LH2 FP for renewable energy (a premise to large mitigation potentials) is considerable. The 210 000 flights, roughly the annual domestic aviation activity in Norway, imply a total energy demand of more than 10 TWh, approximately a tenth of Norway’s current renewable electricity production.^97^ When including fuel for international flights departing from Norway, this number exceeds 30 TWh.^6^ From a societal perspective, it may seem questionable if allocating renewable electricity resources to guarantee continued or rising aviation activity warrants prioritization.^98^ One potential alternative is using biomass feedstocks for SPK fuels, discussed below. Alternatively, limiting energy demand for aviation via efficiency increases, and limiting activity may be a more robust mitigation strategy.^99,100^

Biological feedstocks could substitute carbon from DAC and thereby reduce the renewable energy demand in the production of SPK fuel. However, the environmental and particular climate impacts of biomass production depend on their source.^11,30,101^ Biomass production may compete with other sectors for agricultural land, and related geophysical and biophysical changes to the earth system are relevant for the overall impact.^2,11^ Biological feedstocks remain a potential option, but the topic warrants, given the complexity, further assessment with adequate methods, particularly at the country-level scale.^11^

LH2 and PtL from DAC fuel may offer cobenefits beyond climate change mitigation, e.g., from reduced operational air pollutant formation, or lower land transformation and eutrophication potentials compared to biological feedstock-based SPK fuels or FJF (Supporting Information Table S3^11,102^). However, the large renewable energy needs may also lead to additional environmental burdens, justifying an LCA perspective.

To summarize, we identify fleetwide mitigation potentials of the alternative aviation fuels LH2 and PtL using wind power in the order of 50% compared to fossil jet fuel (GWP100), but those vary with aircraft and flight distance and are subject to uncertainty. By coupling the fuel and emission model AviTeam and LCA, we assess systemwide impacts and show variability in results with regard to the trip distance and highlight a fuel penalty of low-density fuels (LH2). The large share of SLCFs implies that mitigation potentials depend significantly on the time horizon and the implicit weighting of short and long-term impacts. Regardless of the assessed climate metric, LH2 and PtL produced from renewable electricity can perform 30–70% better than FJF. Our results also underline that alternative fuels, while offering some mitigation potential, are neither emission-free nor climate-neutral from a life cycle perspective. Thus, alternatives beyond fuel switching are warranted to align the aviation sector with climate neutrality targets.

## Supporting Information Available

Additional details on the methods and results, data underlying the figures, and life cycle inventory data (ZIP)
Klenner (2024) data for plots (XLSX)
Details of the methods and results (PDF)
Emission metrics for aviation emissions used in this article (XLSX)
