# Supplementary material for: Combining Fleetwide AviTeam Aviation Emission Modeling with LCA Perspectives for an Alternative Fuel Impact Assessment
Source: Environ Sci Technol. 2024 May 16;58(21):9135–46. doi: 10.1021/acs.est.3c08592 (PMC11137866; doi:10.1021/acs.est.3c08592)
Supplement: Supplementary file 3 — es3c08592_si_003.pdf [file es3c08592_si_003.pdf]

Combining fleet-wide AviTeam aviation emission modelling with LCA perspectives for alternative fuel impact assessment

Authors:
Jan Klenner[1*], Marianne T. Lund[2], Helene Muri[1], Anders H. Strømman[1]

Institutions:
[1]: Industrial Ecology Program, Department of Energy and Process Engineering, Norwegian University of Science and Technology (NTNU), 7034 Trondheim, Norway
[2]: Center for International Climate Research (CICERO), 0349 Oslo, Norway

**Corresponding Author:*
Jan Klenner: jan.klenner@ntnu.no

April 9th, 2024

S Supplementary Material

Contents

S.1 AviTeam framework extensions S-4

S.2 Emission indices of synthetic paraffinic kerosene (SPK) S-11

S.3 Life cycle assessment: Extended description S-13

S.4 Benchmarking of fuel production impacts S-14

S.5 Extended results S-16

S.6 ReCiPe2016 (H) - Other categories S-22

S.7 Life cycle inventories S-25

List of Figures

S1 Comparison of fuel demand in unclustered and clustered case. S-6

S2 Comparison of fuel demand in the clustered case with representative aircraft and the larger representative aircraft. S-7

S3 Tank volumes for aircraft clusters S-10

S4 Emission indices of synthetic paraffinic kerosene (SPK). S-12

S5 Comparison of GWP100 for different upstream value chains. S-14

S6 Sensitivity and uncertainty of mitigation potentials. S-16

S7 Mitigation potential of PTL-W and LH2-W using the GWP100 metric and relative to FJF. Mitigation potential of (a) all aircraft in the fleet, (b) jet aircraft, and (c) turboprop and piston aircraft. Solid lines show the mean mitigation potential of PtL-W (blue) and LH2-W (green). Shaded areas show the mitigation potential between the 5% and 95%-quantile of all flights in each 25km group. S-17

S8 GWP20, GWP100, and GTP100 values for all fuels assessed. S-19

S9 CED in fuel production. S-20

S10 Aggregated FJF consumption by clusters. S-21

S11 Aggregated LH2 consumption by clusters. S-21

S12 System stages considered in this well-to-wake study S-26

S13 Fossil jet fuel (FJF) production S-26

S14 Synthesised paraffinic kerosene production in gas-to-liquid pathway S-26

S15 H2 production from alkaline electrolysis S-27

S16 H2 production from NG with ATR or SMR S-27

S17 LH2 production from H2, decentralised S-28

S18 LH2 production, centralised S-28

S19 Synthesised paraffinic kerosene production from H2 and DAC S-29

S20 Synthesised paraffinic kerosene production with solid-oxide electrolysis . . . S-29

List of Tables

S1 The eleven aircraft clusters S-9

S2 GWP and GTP values per MJ-fuel as functional unit S-18

S3 ReCiPe2016 (H) impacts S-23

S4 ReCiPe2016 (H) impacts (continued). S-24

S5 LCI of crude oil and natural gas extraction, Edvard Grieg platform S-30

S6 LCI of crude oil and natural gas extraction, Oseberg platform S-32

S7 LCI of crude oil and natural gas extraction, Statfjord platform S-34

S8 LCI of crude oil and natural gas extraction, Troll platform S-36

S9 LCI of crude oil transport to shore S-38

S11 LCI of natural gas extraction, off-shore, Kristin platform S-39

S10 LCI of fossil jet fuel (FJF) production S-41

S12 LCI of gas transport to shore S-42

S13 LCI of CO_2 production via direct air capture (DAC) with amine-based sorbent. S-42

S14 LCI of direct air capture with calcium carbonate adsorbent. S-43

S15 LCI of CO_2 compression from 1 bar to 25 bar. S-43

S16 LCI of transport, freight, lorry 16−32 metric ton, hydrogen, liquefied. . . . S-44

S.1 AviTeam framework extensions

In this section, we present changes made to the AviTeam version described in Klenner et al. (2022).

Takeoff weight estimation We expand the model by explicitly estimating the takeoff weight for each flight.

$$m_{tow} = m_{oew} + m_{fuel} + m_{payload} \tag{1}$$

Takeoff weight (m_{tow}) is the sum of operating empty weight (m_{oew}), fuel weight (m_{fuel}), and payload ($m_{payload}$) (Eq. 1. The estimation builds on the assumption that each flight takes-off with the amount of fuel recommended by the ICAO guidelines (ICAO, 2015). We apply the recommendations for turbine engines to all flights. For this case study, it is further assumed that the payload carried on each flight equals the maximum payload multiplied by the average cabin factor. In our data, this value is 69% (Statistics Norway, 2023a, 2023b).

$$m_{fuel} = m_{taxi\text{-}out} + m_{trip} + m_{alt.destination} + m_{final} \tag{2}$$

Fuel weight is the sum of several components (Eq. 2). The final fuel reserve (m_{final}) is set to 30min constant flight at $234\,\mathrm{m\,s^{-1}}$ at 450m with the zero-fuel weight ($m_{oew} + m_{payload}$). Alternative destination fuel ($m_{alt.destination}$) is calculated identically but for a flight time of 15 min.

$$m_{lw} = m_{taxi\text{-}in} + m_{alt.destination} + m_{final} \tag{3}$$

The landing weight (m_{lw}) is the sum of taxi-in fuel ($m_{taxi\text{-}in}$), alternative destination fuel and final fuel reserve (Eq. 3). Taxi-in fuel is calculated using the ICAO standard LTO cycle time of taxi-in of 19 min with the corresponding fuel flow of the engine thrust/power setting of 7%. Taxi-out fuel ($m_{taxi\text{-}out}$) is calculated analogously for 7 min.

Trip fuel is then calculated using the Breguet equation, e.g., described in (Lee et al., 2001). For jet aircraft, the following formulation is used.

$$m_{trip} = m_{lw} * (e^{\frac{d*TSFC*g}{v*L/D}} - 1) \tag{4}$$

The airspeed (v) is set to the trip's average speed, the distance (d) to the actual distance flown, lift-to-drag ratio (L/D) is set to the trip's average and is calculated using the BADA 3 model with aircraft reference mass and the standard atmosphere's density. g is the gravity constant. Thrust-specific fuel consumption ($TSFC$) expressed in kg/s is calculated based on the ICAO engine database (ICAO, 2023) by dividing maximal thrust

by maximal fuel flow. In the case of hydrogen, the TSFC is adjusted with the ratio of fossil jet fuels and hydrogen's lower heating value (LHV).

For turboprop and piston aircraft, the Breguet equation's version used is as in Eq. 5:

$$m_{trip} = m_{lw} * (e^{\frac{d*g}{LHV*\rho}} - 1) \tag{5}$$

ρ is the engine's efficiency and assumed as 35%. LHV is the fuel's lower heating value.

Black carbon mass emissions Black carbon (BC) emissions and emission indices are calculated engine-specific and with a non-linear relationship to engine fuel burn. We use the same methodology as Quadros et al. (2022). This approach considers fuel flow, engine types, flight altitude and atmospheric conditions (temperature, pressure, air density). BC emissions for the LTO cycle are calculated with the SCOPE11 approach from Agarwal et al. (2019). The altitude limit for the LTO cycle is set to 3 000 feet (914 m). BC emissions above that altitude are calculated using the correlations described in Peck et al. (2013). BC emissions of turboprop and piston engines are set to 0.03 g kg-kerosene^{-1} above 3 000 feet and zero otherwise.

Aircraft clusters We calculate energy demand and emissions in the operational phase of aircraft with the AviTeam framework (Klenner et al., 2022). The AviTeam uses the Eurocontrol BADA 3 model as aircraft performance model. The Eurocontrol BADA 3 model is described in Nuic et al. (2010), version BADA 3.15 includes a parametrisation for 250 aircraft types. We extract the information of the aircraft type used from the input data, namely Automatic Dependent Surveillance-Broadcast (ADS-B) and flight records provided by Avinor AS.

To simplify the modelling of the LH2 case, we group aircraft into eleven clusters. Criteria for clustering are the aircraft model's year of introduction to the market, size, and engine type. We choose a single aircraft as the representative for each cluster. This representative aircraft is used to simulate all flights of that cluster (Supplementary Tbl. S1). Notably, we only list aircraft that are present in our flight data.

The clustering introduces differences compared to a full-fledged model run with all 250 aircraft types. The R2 between the original fuel burn and clustered fuel burn of all 220,000 flights is 0.53 (Supplementary Fig. S1). The mean absolute percentage error is 17%. The error induced by this clustering to the total energy consumption is -3.6% of the non-clustered baseline fuel burn.

For each cluster, the representative aircraft with its default engine is used. Thus, the clustering also implies differences for each emission species, which may have a different behaviour than fuel burn in the case of emissions that are modelled with non-linear emis-

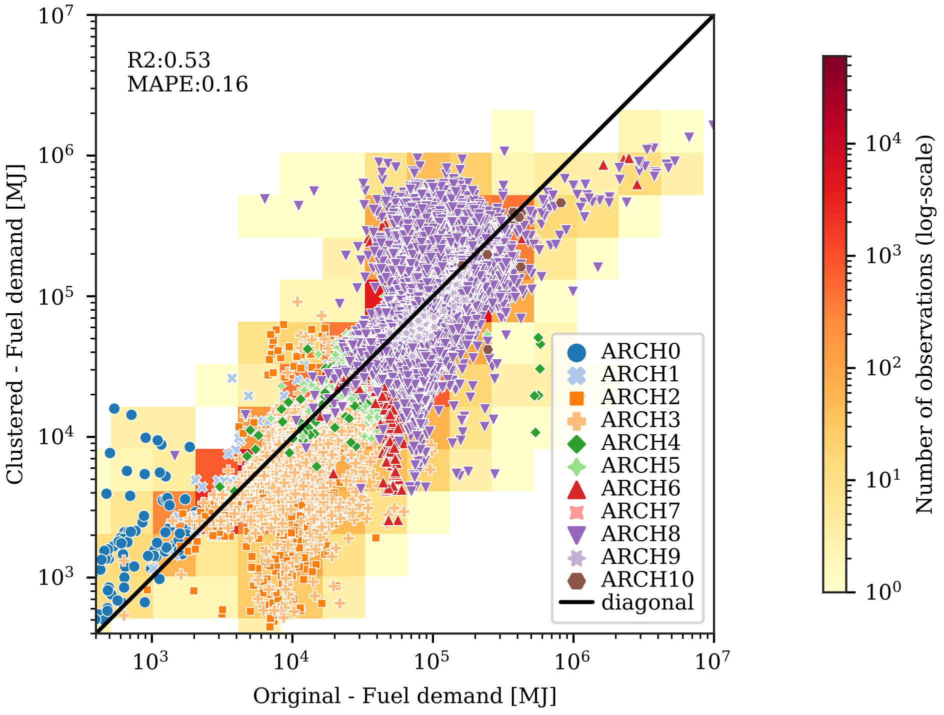

Figure S1: Comparison of fuel demand in unclustered and clustered case.
Fuel demand (MJ) in the unclustered case with 250 aircraft types (original) on x-axis, fuel demand (MJ) in the clustered case with use of representative aircraft (clustered) on the y-axis. Log-scale on both axis. The black diagonal represents a perfect match between the original and clustered fuel demand. Individual flights are shown as a scatterplot, different clusters are represented with different coloured and shaped markers. The background colour indicates the number of observations in the respective area of the plot, from few observations in light yellow to more than 10,000 observations in dark red.

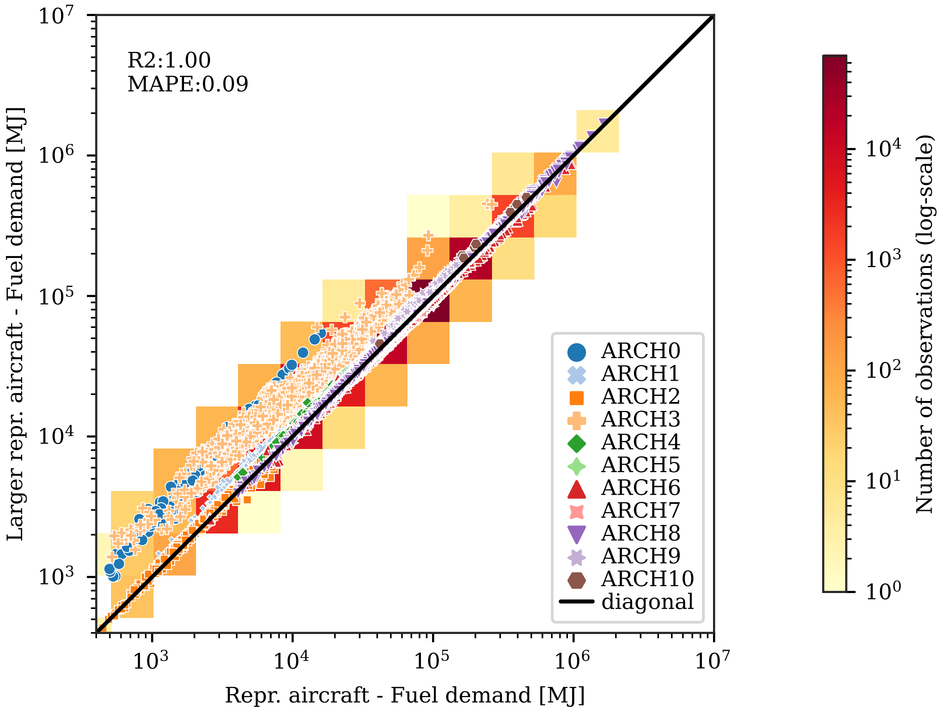

Figure S2: Comparison of fuel demand in the clustered case with representative aircraft and the larger representative aircraft.

Fuel demand (MJ) in the case with representative aircraft (Repr. aircraft) on x-axis, fuel demand (MJ) in the case with larger representative aircraft (Larger repr. aircraft) on the y-axis. Log-scale on both axis. The black diagonal represents a perfect match between both cases. Individual flights are shown as a scatterplot, different clusters are represented with different coloured and shaped markers. The background colour indicates the number of observations in the respective area of the plot, from few observations in light yellow to more than 10,000 observations in dark red.

sion indices. These are -10.2% for NO_x, -18.6% for HC, +7.5% for CO, and +1.1% for BC emissions compared to the baseline.

The case of liquid hydrogen aircraft In the case of liquid hydrogen fuel, the additional tank space required for hydrogen tanks is taken into consideration. For each cluster, a larger representative aircraft is chosen. The fuselages' volume difference is considered as additional tank space available for hydrogen tanks, following design ideas that fit hydrogen tanks inside the fuselage as presented in Snyder et al. (2009) and Verstraete et al. (2010). The larger representative aircraft is preferably from the same aircraft cluster to guarantee a comparable performance. Supplementary Tbl. S1 shows the original aircraft tank volume, the aircraft type chosen for LH2 flight, and the hypothetical tank volume for the same amounts of seats given the larger variant chosen.

The minimal tank requirement for each aircraft cluster is calculated by taking the 95%-quantile of the fuel consumption distribution in each aircraft cluster using FJF. Then, 30 min of cruise fuel, 5% reserve fuel, and 15 min as proxy for fuel to the next airport as specified by the ICAO regulations (https://skybrary.aero/articles/fuel-regulations) are added. The final values as minimal LH2 tank capacity in m^3 and MJ are given in Figure S3. In addition, Figure S3 shows the original tank volume of the smaller aircraft in m^3 and MJ, and the tank capacity of the larger representative aircraft assuming that the fuselage volume difference can be fully used for additional LH2 tanks.

Table S1: The eleven aircraft clusters.
Columns in order: Cluster name, Cluster description, Aircraft used as representative of the cluster, Aircraft in cluster (only observed), Tank size of representative aircraft in m^3, Larger version of representative aircraft used in hydrogen case, Tank volume for the use of the larger representative aircraft in m^3.

Cluster	Description	Repr. aircraft	Aircraft	Tank [m^3]	Large repr. aircraft	Large tank [m^3]	No. seats
ARCH0	Piston aircraft	PA44	S22T, SR22, PA31, DA42, DA40, P28T, PA34, C206, P28A, DA62, C172	0.4	PA31	2	3
ARCH1	Smallest turboprop	BE20	BE20, PAY3, PC12, C208, D228, P46T, BE30, KODI, P180, SW4, BE9L, B350	2	B350	3	10
ARCH2	Medium turboprop	DH8A	DH8A, SF34, E120, F27, F50	3	DH8C	23	38
ARCH3	Largest turboprop	DH8C	AT45, ATP, DH8C, DH8D	7	DH8D	44	50
ARCH4	Smaller business jets, <15t MTOW	C680	C56X, E55P, C510, C680, C525, E50P, BE40, EA50, LJ35, C25A, C550, H25B, C560, LJ45, HDJT, C25C	6	G280	39	9
ARCH5	Larger business jets, smallest narrow-body aircraft, >15t MTOW	CRJ9	E135, E35L, E145, CL60, CRJ9, G280, CRJ2, GLEX, F2TH, RJ1H, FA7X, GL5T, F900, GLF5, GLF2, CL30	11	CRJX	28	78
ARCH6	Smaller narrow-body aircraft	E75L	E170, E75L, E190, E290	12	E290	43	82
ARCH7	Larger narrow-body aircraft, oldest generation	B735	B735, B734, B733	20	B734	80	127
ARCH8	Larger narrow-body aircraft, mid-aged generation	B738	B737, A319, A320, A321, B738, B736	26	B739	55	170
ARCH9	Larger narrow-body aircraft, newest generation	A20N	A20N, B38M	27	A21N	184	196
ARCH10	Wide-body aircraft	A332	B772, B763, B762, A332, A306, B744	85	A333	248	345

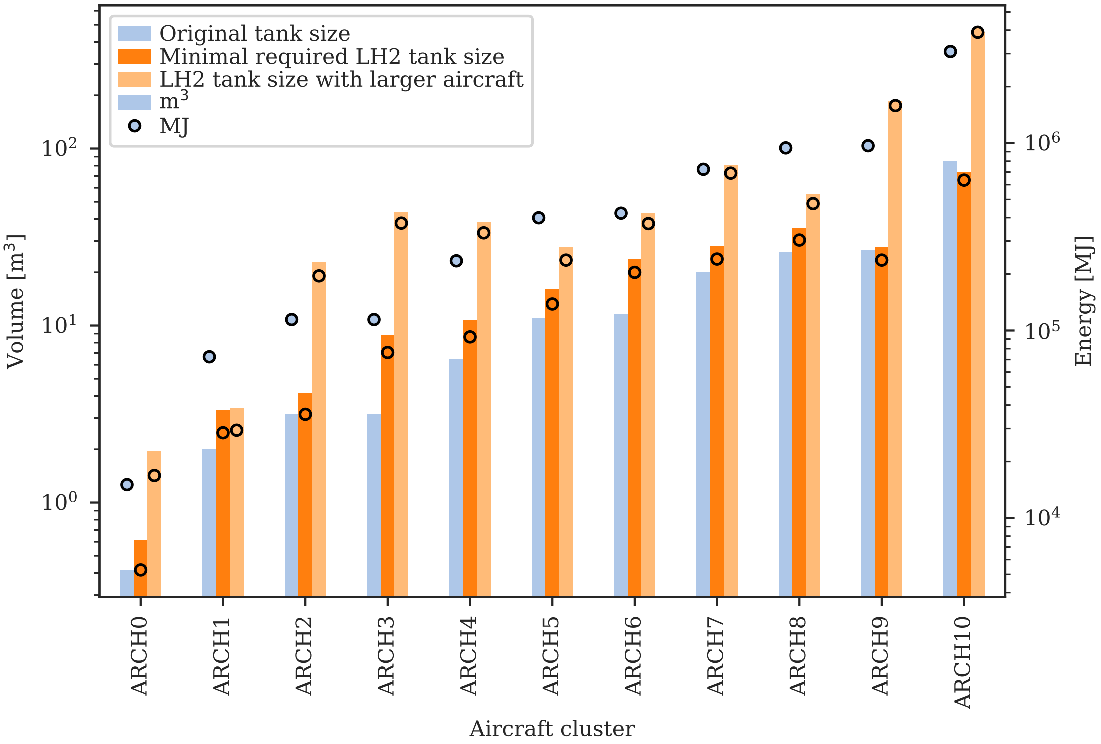

Figure S3: Tank volumes for aircraft clusters.
Tank volumes in m^3 (bars) and MJ-fuel (circles). Original tank size is the current tank size of the cluster's representative aircraft. Minimal required LH2 tank size describes tank size needed to fulfil 95% of all missions in the current dataset. The value is the 95%-quantile of the current mission distribution of energy demand in MJ. The LH2 tank size with larger aircraft describes the volume available for LH2 tanks adding the additional volume of using a larger aircraft to the original tank size. The extended aircraft's tank is large enough if the LH2 tank size with larger aircraft is larger than the minimal required LH2 tank size, which is met for all clusters. Assumed y-axes in log-scale. Assumed energy densities are FJF: 36 204 MJ m^{-3} and LH2: 8 591 MJ m^{-3}.

S.2 Emission indices of synthetic paraffinic kerosene (SPK)

To model alternative fuels, we estimate gaseous and particulate emissions of aircraft fuelled with SPK. Emissions (in kg) can be expressed as the product of emission indices (in kg emission per MJ-fuel) and fuel burn (in MJ-fuel). In this process, we focus on jet engines as they are responsible for the largest share of emissions. We do not have data that suggest that the response of turboprop engines to a fuel switch will be significantly different to the response of jet engines. It is also commonly accepted that jet engine emissions can be used to extrapolate turboprop engine emissions, evidencing a similar combustion behaviour (Filippone et al., 2018).

Emission indices of SPK synthesised via the Fischer-Tropsch (FT) or hydroprocessed esters and fatty acids (HEFA) process have been studied in different laboratory, and field experiments. We understand that the chemical composition of the final product is subject to the fine-tuning of the FT process (or alternatives) and subsequent refinement. Thus, we do not distinguish between low-aromatic SPK produced from different processes and feedstocks. Most studies analyse different mixing ratios of conventional kerosene and alternative aviation fuels. As emission indices do not necessarily vary linearly with the mixing ratio, we consider only neat alternative fuels or mixtures of more than 90% in the analysis. We include several observations from one study if observations data are presented separately and we include observations from jet engines designed for helicopters and auxiliary power units.

The following studies are used as references for NO_x emissions: (Cain et al., 2013; Corporan et al., 2007; Corporan et al., 2010; Corporan et al., 2011; Harper et al., 2022; Khandelwal et al., 2019; Moore et al., 2017; Schripp et al., 2018; Snijders et al., 2011).

The following studies are used as references for CO emissions: (Cain et al., 2013; Corporan et al., 2010; Corporan et al., 2011; DeWitt et al., 2008; Harper et al., 2022; Khandelwal et al., 2019; Li-Jones et al., 2007; Moore et al., 2017; Schripp et al., 2018; Snijders et al., 2011; Timko et al., 2011; Undavalli et al., 2022).

The following studies are used as references for HC emissions: (Cain et al., 2013; Corporan et al., 2011; DeWitt et al., 2008; Khandelwal et al., 2019; Li-Jones et al., 2007; Undavalli et al., 2022).

The following studies are used as references for BC emissions: (Cain et al., 2013; Corporan et al., 2007; Corporan et al., 2010; Corporan et al., 2011; DeWitt et al., 2008; Drozd et al., 2012; Harper et al., 2022; Khandelwal et al., 2019; Li-Jones et al., 2007; Snijders et al., 2011; Undavalli et al., 2022).

The following studies are used as references for OC emissions: (Corporan et al., 2010; Drozd et al., 2012; Moore et al., 2015; Williams et al., 2012)

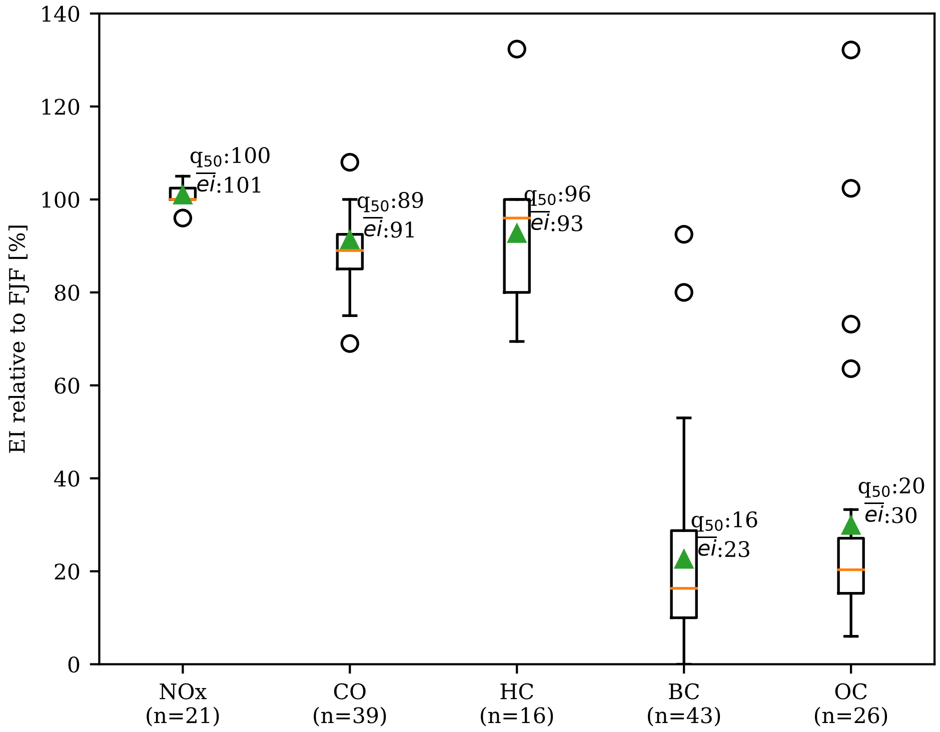

Figure S4: Emission indices of synthetic paraffinic kerosene (SPK).
Boxplots for the species NO_x, CO, HC, BC, and OC. Emission index for different species relative to FJF emissions in the same study on y-axis in %. n indicates the number of datapoints for each species. q_{50} is the median, ei the mean. The boxplot shows medians (orange lines), means (green triangles), interquartile ranges (edges of box), 1.5-interquartile ranges as the end of whiskers and outliers (empty circles).

S.3 Life cycle assessment: Extended description

This section includes a condensed description of LCA goal and scope.

Goal definition The primary goal of this study is to assess the fleet-wide climate impacts of using different aviation fuels in a network with 210 000 shorter-haul flights. The underlying motivation is to complement existing literature on aviation fuels with insights that can be derived using our combination of the high-resolution aviation fuel burn and emission model AviTeam and the life-cycle perspective. Using the AviTeam model, we can i) endogenise the implications of a fuel switch on the entire fleet's energy demand, ii) point to variability in the mitigation potential of alternative fuels depending on flight distance, and iii) provide detailed aviation emission estimates to discuss the balance of short-lived and long-lived climate forcings and the mitigation potential of alternative aviation fuels. We compare ten fuel production pathways and include an analysis of sensitivity to key assumptions in our work.

Scope The system studied refers to a set of 210 000 flights in 2019 using the 2019 fleet technology in the study area and different technology assumptions for alternative fuels. The functional unit compared is the transport work provided measured in seat-kilometres and assessed for each flight individually. As previously mentioned, cargo flights' work is converted to seat-kilometres using the indicated seating capacity.

System boundaries of this well-to-wake (WTW) assessment are visualised in Figure S12. The foreground system comprises resource extraction, transport, conversion to fuel, fuel transport to and refilling at the airport, and the fuel combustion modelled with the AviTeam. The background system includes airport and aircraft infrastructure, and other the production of other process inputs as indicated in the LCI tables (Supplementary Tables S5–S16). Infrastructure and end-of-life inventories are included in background inventories where available. The background system is modelled with the ecoinvent 3.8 (cutoff) database (Wernet et al., 2016). For all extraction and refinery processes, energy-content-based allocation is chosen.

The impact categories selected and the methodology of impact assessment are described in more detail in the methods section. Climate change impacts are measured in GWP and GTP using newly derived emission metrics for aviation emissions in the operational phase and the GWP and GTP metrics from Myhre et al. (2013) to the remaining phases. Further, the metric of cumulative energy demand (CED) is used and results are provided for ReCiPe 2016 Hierarchist Midpoint indicators.

S.4 Benchmarking of fuel production impacts

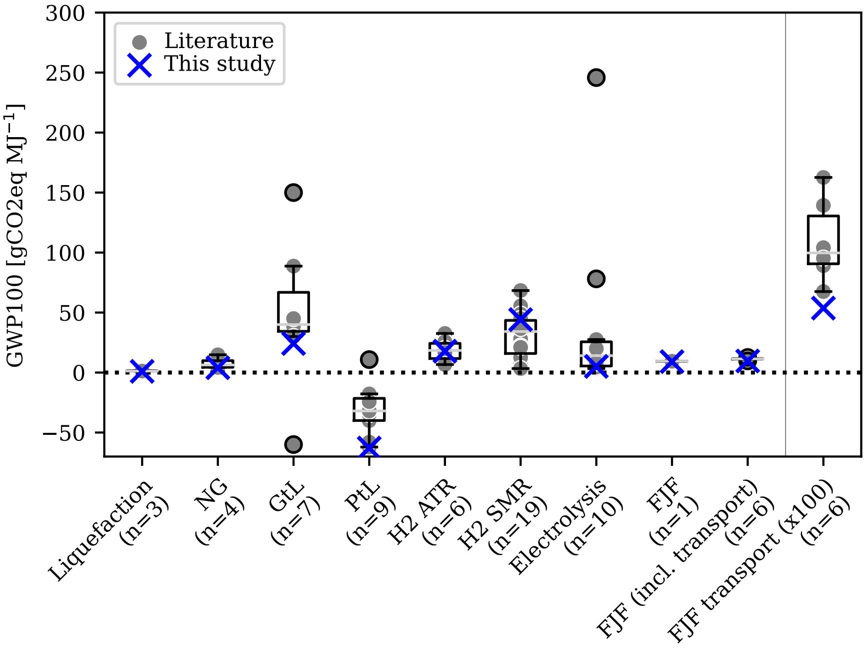

Figure S5: Comparison of GWP100 for different upstream value chains.
Values of GWP100 in $kgCO_2eq$ MJ $^{-1}$ on y axis. This study marked with blue x's, values from literature as grey circles. Literature sources are described in the paragraph below. NG: Natural gas; GtL: Gas-to-liquid fuel; H2-ATR: H2 from auto-thermal reforming; H2-SMR: Hydrogen from steam methane reforming; Values for GtL, PtL, H2, and FJF for fuel at market or airports. Values for liquefaction at liquefaction facility and natural gas at market (NG). Values for transport scaled by a factor 100 for better comparability of results. Number of reference values in literature in parenthesis.

To benchmark our results for the fuel production pathway modelling, we compare the GWP100 of our results with those in literature (Supplementary Figure S5). The usage of low-impact wind power explains lower GWP100 emissions in PtL production and electrolysis. FJF transport impacts modelled are lower than in other data sets in ecoinvent (Wernet et al., 2016), but their relative importance in the overall emissions is very small (Supplementary Figure S5).

We use the following sources for the GWP impacts: Liquefaction: (Akhtar et al., 2021; Kolb et al., 2022); Natural gas extraction and distribution: (Skone et al., 2016; Wernet et al., 2016); Gas-to-liquid fuel: (Bengtsson et al., 2011; Elgowainy et al., 2012; Forman et al., 2011; van der Giesen et al., 2014); Power-to-liquid fuel: (Ballal et al., 2023; Micheli et al., 2022; van der Giesen et al., 2014; Yugo et al., 2021) H2 from auto-thermal reforming: (Antonini et al., 2020; Hydrogen Council, 2021; Oni et al., 2022; Rooijers et al., 2018); H2 from steam methane reforming: (Antonini et al., 2020; Baldino et al., 2020; Ewing et al., 2020; Hydrogen Council, 2021; Longden et al., 2022; Oni et al., 2022; Rooijers et al., 2018); Electrolysis: (Bareiß et al., 2019; Cetinkaya et al., 2012; Matzen et al., 2015; van der Giesen et al., 2014; Zhang et al., 2022; Zhao et al., 2020); Fossil jet fuel: (Wernet et

al., 2016); Fossil jet fuel (incl. transport): (Wernet et al., 2016); FJF transport: (Wernet et al., 2016).

One aspect not included in the fuel production chain of LH2 is the quantification of climatic impacts of H_2-leakages. Recent quantification sets the GWP of H_2-leakages close to ground at 10.9 (6.4 − 15.3) kgCO$_2$eq kgH$_2^{-1}$ (Ocko et al., 2022; Warwick et al., 2022). In our case, this implies a small additional burden of 1 gCO$_2$eq seat-km^{-1} (GWP100). With larger H_2 losses or venting, one may have to consider the effect of H_2 emissions explicitly, c.f. Ocko et al. (2022).

S.5 Extended results

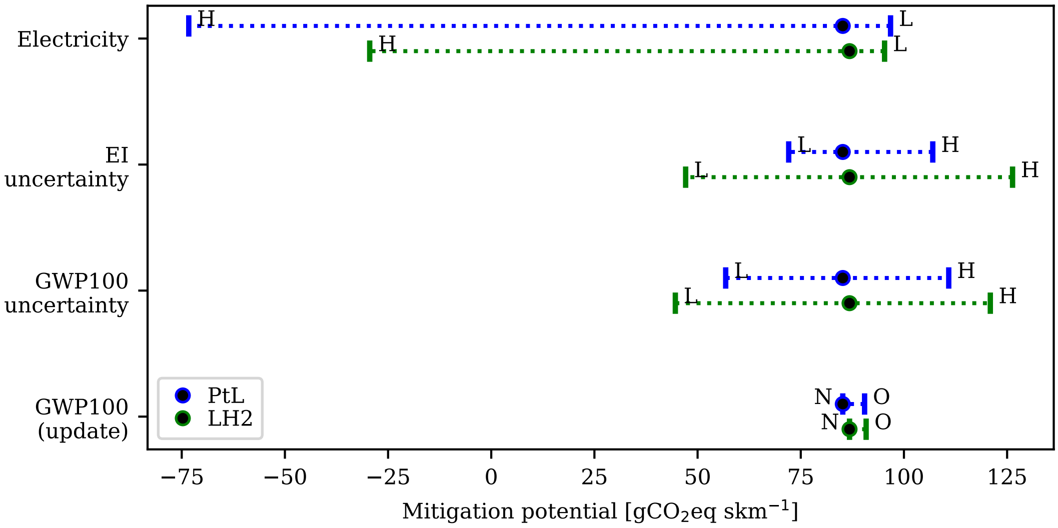

Figure S6: Sensitivity and uncertainty of mitigation potentials.

This study's best estimates (black circles), and range between lowest and highest mitigation potential (bars with whiskers).

First row: Sensitivity to the electricity mix's GWP. Electricity GWPs 0 gCO$_2$eq kWh^{-1} (L), 13 gCO$_2$eq kWh^{-1} (best estimate), and 200 gCO$_2$eq kWh^{-1} (H).

Second row: Uncertainty in emission indices and contrail cirrus formation of alternative fuels. Estimates for low reductions (L), best estimates, and high reductions of emissions (H) relative to FJF as provided in Table 1. Contrail cirrus ranges of FJF's contrail cirrus 20% to 80% for PtL, and 26% to 130% for LH2.

Third row: Uncertainty of GWP estimates of SLCF, using 5% quantile (L), best, and 95% quantile (H) estimates of radiative forcings as the basis for GWP calculation.

Fourth row: Mitigation potentials under the assumption of using previous GWP metrics from (Lund et al., 2017) (O) or newly derived metrics (N).

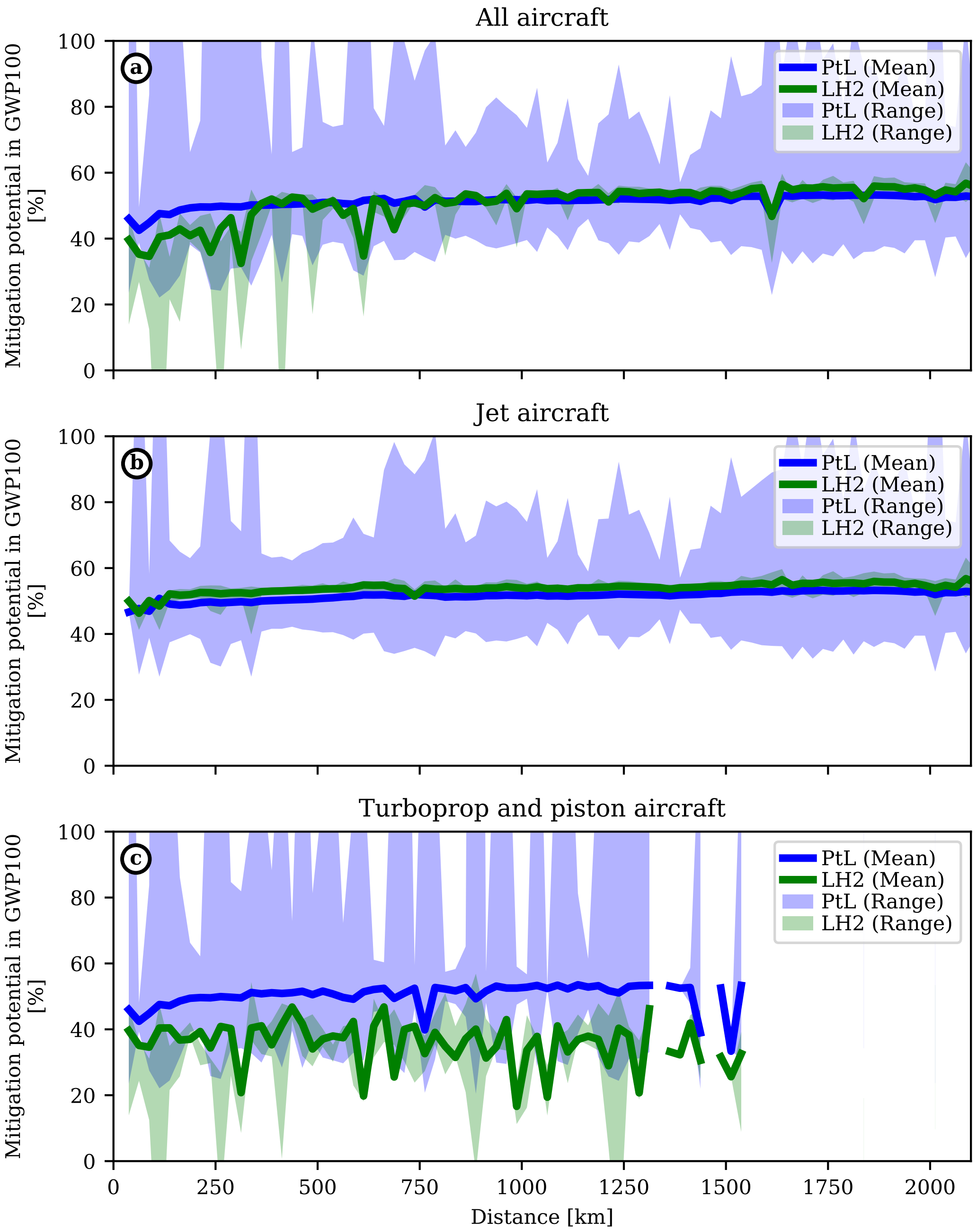

Figure S7: Mitigation potential of PTL-W and LH2-W using the GWP100 metric and relative to FJF. Mitigation potential of (a) all aircraft in the fleet, (b) jet aircraft, and (c) turboprop and piston aircraft. Solid lines show the mean mitigation potential of PtL-W (blue) and LH2-W (green). Shaded areas show the mitigation potential between the 5% and 95%-quantile of all flights in each 25km group.

Table S2: GWP and GTP values per MJ-fuel as functional unit. Operational impacts are shown by individual emissions marked with (O), fuel and aircraft and airport emissions in aggregated form (A). Fuel production impacts of PtL fuel exclude CO_2 adsorbed in DAC, which is listed separately as CO_2(DAC). GWP values in gCO_2eq MJ-fuel^{-1}.

Unit:	GWP20			GWP100			GTP100		
gCO_2eq MJ-fuel^{-1}	FJF	PtL	LH2	FJF	PtL	LH2	FJF	PtL	LH2
CO_2 (O)	73.9	73.9		73.9	73.9		73.9	73.9	
CO_2 (DAC)		−73.9			−73.9			−73.9	
H_2O (O)	11.5	11.5	31.1	2.9	2.9	7.8	0.5	0.5	1.4
Contrail cirrus (O)	147.9	85.8	130.6	51.8	30.0	45.7	8.9	5.1	7.8
OC (O)	-2.2	-0.5		-0.6	-0.1		-0.1	-0.0	
CO (O)	1.2	1.1		0.5	0.5		0.3	0.2	
NO_x (O)	76.9	76.9	30.1	12.9	12.9	5.0	1.7	1.7	0.7
SO_x (O)	-21.9			-5.9			-1.1		
BC (O)	5.1	1.3		1.4	0.3		0.3	0.1	
Airport+Aircraft (A)	6.3	6.3	6.1	5.7	5.7	5.5	5.5	5.5	5.3
Fuel (A)	10.1	26.8	10.6	9.7	22.8	8.9	9.5	21.1	8.2
Total	308.9	209.1	208.4	152.2	75.0	72.9	99.3	34.3	23.3

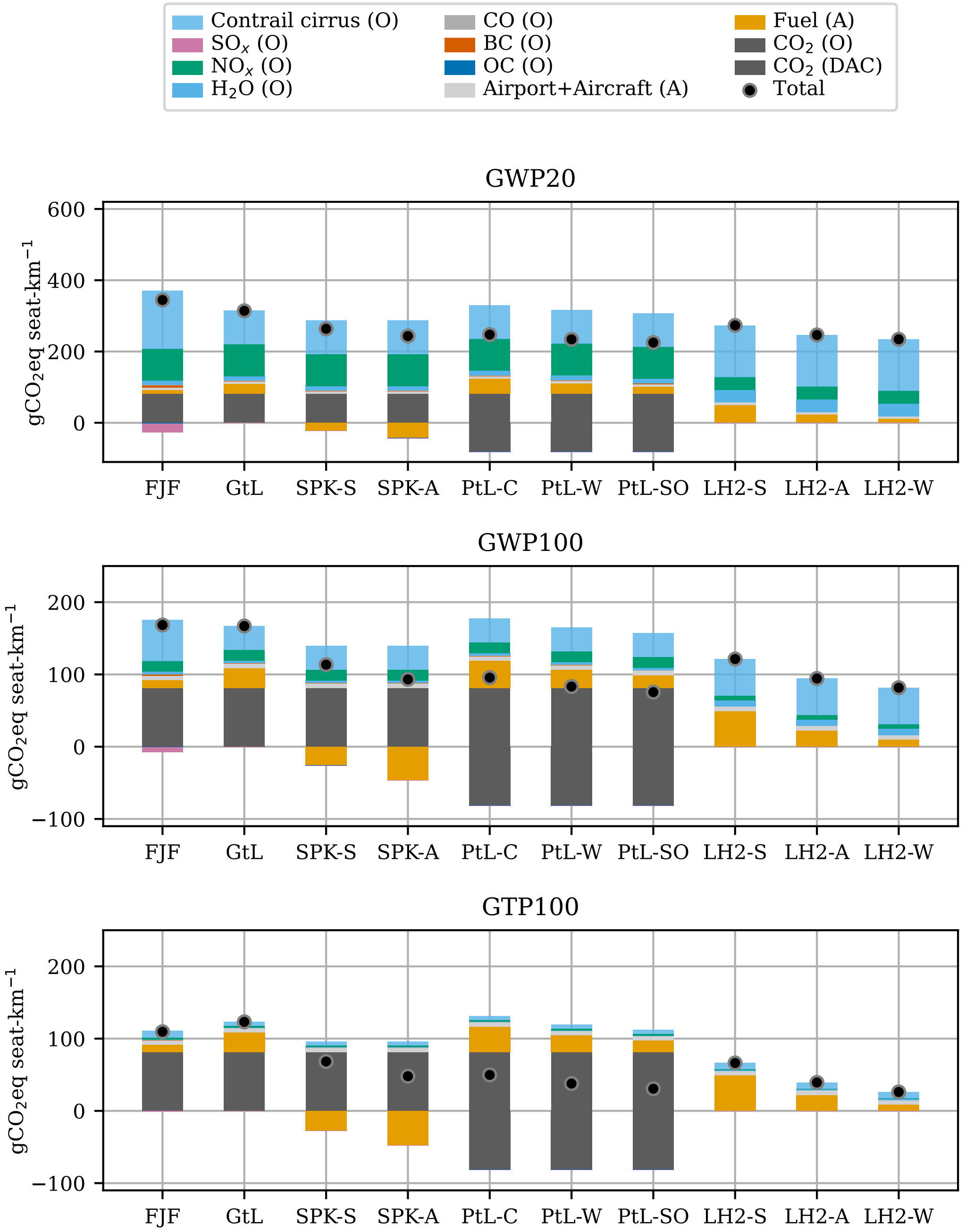

Figure S8: GWP20, GWP100, and GTP100 values for all fuels assessed. y-axis in gCO_2eq per seat-km. FJF: Fossil jet fuel (kerosene); GtL: Gas-to-liquid fuel; LH2-SMR: LH2 from SMR; LH2-ATR: LH2 from ATR; SPK-S: Synthetic paraffinic kerosene from SMR and alkaline electrolysis; SPK-A: Synthetic paraffinic kerosene from ATR and alkaline electrolysis; PTL-CA: PTL from DAC with CaCO3 adsorbent and alkaline electrolysis; PTL-AA: PTL from DAC with amine-based adsorbent and alkaline electrolysis; PTL-SO: PTL from DAC with amine-based adsorbent and SOEC; LH2-A: LH2 from alkaline electrolysis. Impacts are divided into operational impacts, shown by individual emissions marked with (O), and other impacts with aggregated impacts across all emissions marked with (A) and displayed with a gridded filling. CO_2 captured in DAC is accounted for separately as CO_2 (DAC). Positive and negative contributions are stacked separately. Total values as the sum of positive and negative contributions are marked by black circles.

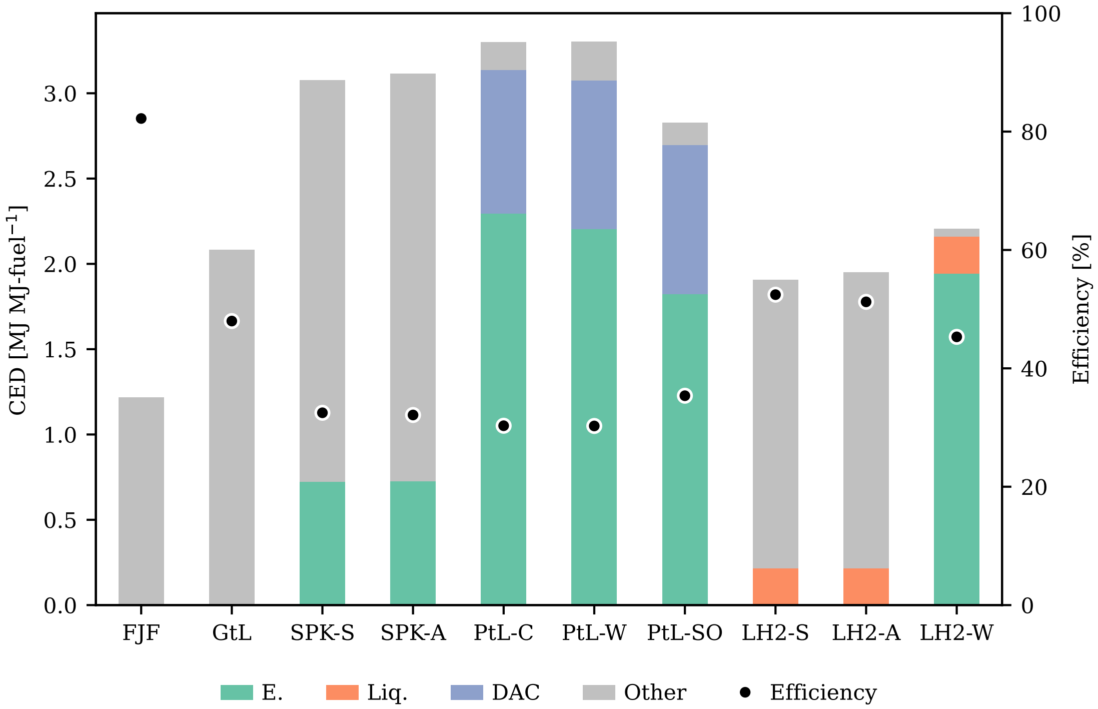

Figure S9: CED in fuel production.

CED in megajoule (MJ) per MJ-fuel (lower heating value) on y-axis. Different fuel are on the x-axis. FJF: Fossil jet fuel (kerosene); GtL: Gas-to-liquid fuel; SPK-S: Synthetic parafinic kerosene from SMR and alkaline electrolysis; SPK-A: Synthetic parafinic kerosene from ATR and alkaline electrolysis; PTL-C: PTL from DAC with CaCO3 and alkaline electrolysis and wind energy; PTL-W: PTL from DAC with MEA and alkaline electrolysis and wind energy; PTL-SO: PTL from DAC with MEA and SOEC; LH2-S: LH2 from SMR; LH2-A: LH2 from ATR; LH2-W: LH2 from alkaline electrolysis and wind energy.

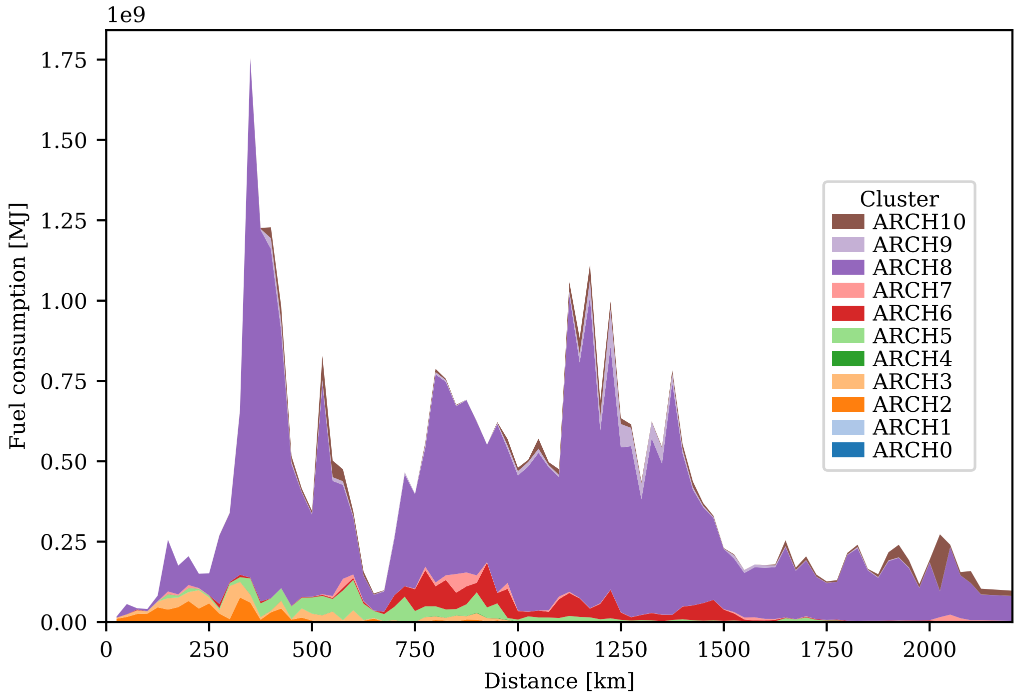

Figure S10: Aggregated FJF consumption by clusters.
Trip distance in km on x-axis. Trip fuel (energy demand without fuel production) in MJ on y-axis. Clusters in different colours.

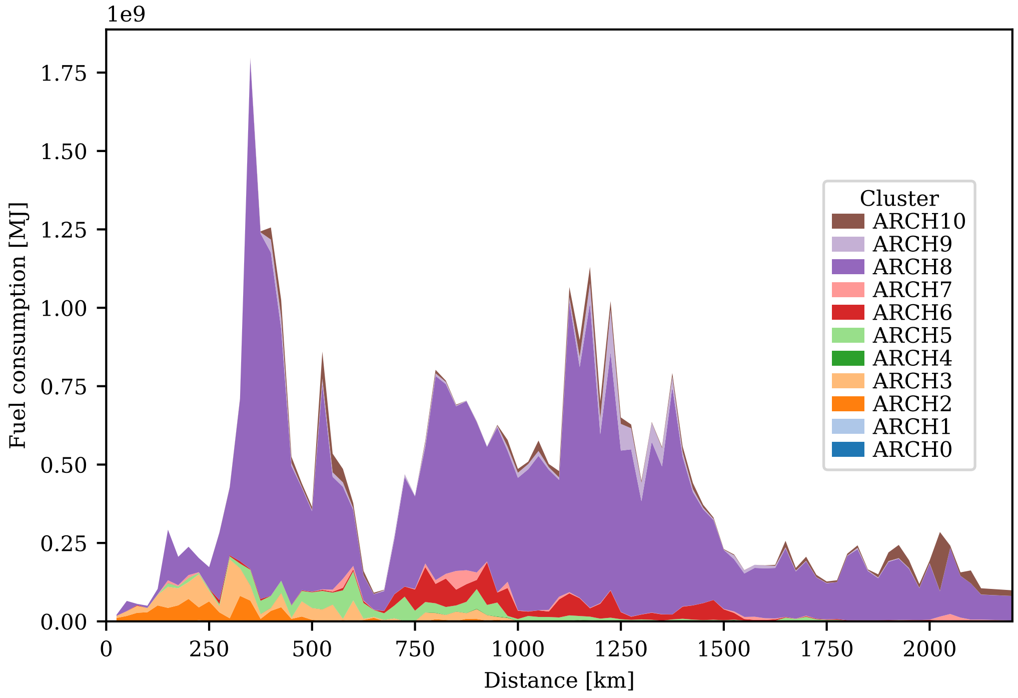

Figure S11: Aggregated LH2 consumption by clusters.
Trip distance in km on x-axis. Trip fuel (energy demand without fuel production) in MJ on y-axis. Clusters in different colours.

S.6 ReCiPe2016 (H) - Other categories

We further present results for the characterization of our inventories with the ReCiPe2016 (H) method (Huijbregts et al., 2017). We wish to comment that the characterisation factors used by ReCiPe2016 may not be suitable for aviation emissions as they are generally developed for a mix of emissions (usually close to the surface and for regions different from Norway). Although provided here, we recommend to consider the characterisation of operational emissions with ReCiPe2016 with caution. Emissions in the climb, cruise, and approach stages are considered little harmful in the categories of particulate matter formation and photochemical oxidation formation (compare Cox et al. (2018)). The assumption is that those emissions, when occurring in higher layers of the atmosphere, are subject to atmospheric reactions and will not circulate into the planetary boundary layer before the end of their lifetime. Thus, their characterisation factors are set to zero.

Table S3: ReCiPe2016 (H) impacts

FJF: Fossil jet fuel (kerosene); GtL: Gas-to-liquid fuel; SPK-S: Synthetic paraffinic kerosene from SMR and alkaline electrolysis; SPK-A: Synthetic paraffinic kerosene from ATR and alkaline electrolysis; PTL-C: PTL from DAC with CaCO3 and alkaline electrolysis; PTL-W: PTL from DAC with MEA and alkaline electrolysis; PTL-SO: PTL from DAC with MEA and SOEC; LH2-S: LH2 from SMR; LH2-A: LH2 from ATR; LH2-W: LH2 from alkaline electrolysis; TAP: Terrestrial acidification potential; POFP: Photochemical oxidant formation potential; PMFP: particulate matter formation potential; MDP: Metal depletion; HTP: Human toxicity potential; METP: Marine ecotoxicity.

Method	Category	FJF	GtL	SPK-S	SPK-A	PtL-C	PTL-W	PtL-SO	LH2-S	LH2-A	LH2-W
TAP	Fuel (A)	4.70e-05	2.14e-05	1.22e-04	1.34e-04	1.73e-04	1.24e-04	9.65e-05	4.32e-05	5.86e-05	4.72e-05
	Airport+Aircraft (A)	2.50e-05	2.50e-05	2.50e-05	2.50e-05	2.50e-05	2.50e-05	2.50e-05	2.52e-05	2.52e-05	2.52e-05
	NO_x (O)	6.99e-05	6.99e-05	6.99e-05	6.99e-05	6.99e-05	6.99e-05	6.99e-05	2.87e-05	2.87e-05	2.87e-05
	SO_x (O)	3.09e-05									
POFP	Fuel (A)	6.14e-05	4.12e-05	1.09e-04	1.29e-04	1.22e-04	1.01e-04	7.39e-05	4.68e-05	7.34e-05	3.99e-05
	Airport+Aircraft (A)	2.78e-05	2.78e-05	2.78e-05	2.78e-05	2.78e-05	2.78e-05	2.78e-05	2.79e-05	2.79e-05	2.79e-05
	NO_x (O)	1.95e-05	1.95e-05	1.95e-05	1.95e-05	1.95e-05	1.95e-05	1.95e-05	8.07e-06	8.07e-06	8.07e-06
	CO (O)	5.59e-07	5.03e-07	5.03e-07	5.03e-07	5.03e-07	5.03e-07	5.03e-07			
	SO_x (O)	1.05e-07									
PMFP	Fuel (A)	1.74e-05	1.07e-05	8.05e-05	8.51e-05	1.05e-04	8.85e-05	5.82e-05	2.29e-05	2.91e-05	3.51e-05
	Airport+Aircraft (A)	9.16e-06	9.16e-06	9.16e-06	9.16e-06	9.16e-06	9.16e-06	9.16e-06	9.22e-06	9.22e-06	9.22e-06
	NO_x (O)	4.28e-06	4.28e-06	4.28e-06	4.28e-06	4.28e-06	4.28e-06	4.28e-06	1.77e-06	1.77e-06	1.77e-06
	SO_x (O)	2.59e-07									
MDP	Fuel (A)	2.43e-04	6.53e-04	1.85e-02	1.85e-02	2.18e-02	2.26e-02	1.34e-02	3.88e-03	3.91e-03	9.31e-03
	Airport+Aircraft (A)	4.00e-04	4.00e-04	4.00e-04	4.00e-04	4.00e-04	4.00e-04	4.00e-04	4.02e-04	4.02e-04	4.02e-04
HTP	Fuel (A)	6.56e-04	8.27e-04	1.66e-02	1.67e-02	2.70e-02	1.82e-02	1.44e-02	5.48e-03	5.62e-03	7.81e-03
	Airport+Aircraft (A)	3.60e-03	3.60e-03	3.60e-03	3.60e-03	3.60e-03	3.60e-03	3.60e-03	3.60e-03	3.60e-03	3.60e-03
METP	Fuel (A)	7.96e-05	1.34e-04	3.49e-03	3.50e-03	4.78e-03	4.80e-03	3.80e-03	9.05e-04	9.11e-04	2.65e-03
	Airport+Aircraft (A)	2.56e-04	2.56e-04	2.56e-04	2.56e-04	2.56e-04	2.56e-04	2.56e-04	2.58e-04	2.58e-04	2.58e-04

Table S4: ReCiPe2016 (H) impacts (continued).

MEP: Marine eutrophication; FDP: Fossil resource scarcity IRP: Ionising radiation; ODP: Ozone depletion potential; FETP: Freshwater ecotoxicity; ALOP: Agricultural land occupation NLTP: Natural land transformation; ULOP: Urban land occupation; WDP: Water depletion potential; FEP: Freshwater eutrophication potential.

Method	Category	FJF	GtL	SPK-S	SPK-A	PtL-C	PTL-W	PtL-SO	LH2-S	LH2-A	LH2-W
MEP	Fuel (A)	1.74e-05	1.22e-05	3.29e-05	4.08e-05	4.01e-05	3.01e-05	2.28e-05	1.43e-05	2.47e-05	1.18e-05
	Airport+Aircraft (A)	5.85e-06	5.85e-06	5.85e-06	5.85e-06	5.85e-06	5.85e-06	5.85e-06	5.88e-06	5.88e-06	5.88e-06
	NO_x (O)	4.85e-05	4.85e-05	4.85e-05	4.85e-05	4.85e-05	4.85e-05	4.85e-05	1.99e-05	1.99e-05	1.99e-05
FDP	Fuel (A)	3.21e-02	5.49e-02	3.37e-02	3.40e-02	1.05e-02	7.10e-03	5.22e-03	3.75e-02	3.79e-02	2.67e-03
	Airport+Aircraft (A)	2.29e-03	2.29e-03	2.29e-03	2.29e-03	2.29e-03	2.29e-03	2.29e-03	2.31e-03	2.31e-03	2.31e-03
IRP	Fuel (A)	8.62e-03	1.57e-04	2.73e-03	2.88e-03	1.04e-02	1.62e-03	1.25e-03	1.99e-03	2.19e-03	5.60e-04
	Airport+Aircraft (A)	2.05e-03	2.05e-03	2.05e-03	2.05e-03	2.05e-03	2.05e-03	2.05e-03	2.05e-03	2.05e-03	2.05e-03
ODP	Fuel (A)	4.71e-10	2.15e-09	2.21e-09	2.23e-09	1.84e-09	1.28e-09	9.22e-10	1.65e-09	1.68e-09	4.70e-10
	Airport+Aircraft (A)	6.88e-10	6.88e-10	6.88e-10	6.88e-10	6.88e-10	6.88e-10	6.88e-10	6.99e-10	6.99e-10	6.99e-10
FETP	Fuel (A)	3.40e-05	4.75e-05	3.81e-03	3.81e-03	5.30e-03	5.36e-03	4.27e-03	9.35e-04	9.41e-04	2.99e-03
	Airport+Aircraft (A)	2.81e-04	2.81e-04	2.81e-04	2.81e-04	2.81e-04	2.81e-04	2.81e-04	2.84e-04	2.84e-04	2.84e-04
TETP	Fuel (A)	7.53e-07	7.30e-07	3.84e-06	3.85e-06	3.95e-06	3.73e-06	2.75e-06	1.20e-06	1.21e-06	1.62e-06
	Airport+Aircraft (A)	3.78e-07	3.78e-07	3.78e-07	3.78e-07	3.78e-07	3.78e-07	3.78e-07	3.80e-07	3.80e-07	3.80e-07
ALOP	Fuel (A)	2.34e-05	3.46e-05	1.21e-03	1.25e-03	1.95e-03	9.52e-04	6.15e-04	6.78e-04	7.21e-04	3.42e-04
	Airport+Aircraft (A)	4.67e-04	4.67e-04	4.67e-04	4.67e-04	4.67e-04	4.67e-04	4.67e-04	4.68e-04	4.68e-04	4.68e-04
NLTP	Fuel (A)	2.01e-05	3.40e-05	2.09e-05	2.11e-05	4.79e-06	3.47e-06	2.54e-06	2.40e-05	2.43e-05	1.18e-06
	Airport+Aircraft (A)	2.94e-06	2.94e-06	2.94e-06	2.94e-06	2.94e-06	2.94e-06	2.94e-06	2.96e-06	2.96e-06	2.96e-06
ULOP	Fuel (A)	1.23e-04	1.42e-04	1.11e-03	1.12e-03	1.52e-03	1.59e-03	1.29e-03	2.80e-04	2.83e-04	9.97e-04
	Airport+Aircraft (A)	4.64e-04	4.64e-04	4.64e-04	4.64e-04	4.64e-04	4.64e-04	4.64e-04	4.64e-04	4.64e-04	4.64e-04
WDP	Fuel (A)	2.48e-06	5.00e-06	1.91e-04	1.92e-04	3.08e-04	2.25e-04	1.28e-04	1.11e-04	1.12e-04	1.56e-04
	Airport+Aircraft (A)	1.12e-04	1.12e-04	1.12e-04	1.12e-04	1.12e-04	1.12e-04	1.12e-04	1.12e-04	1.12e-04	1.12e-04
FEP	Fuel (A)	2.69e-07	4.70e-07	9.88e-06	1.00e-05	2.46e-05	1.09e-05	8.36e-06	3.03e-06	3.20e-06	4.47e-06
	Airport+Aircraft (A)	3.86e-06	3.86e-06	3.86e-06	3.86e-06	3.86e-06	3.86e-06	3.86e-06	3.87e-06	3.87e-06	3.87e-06

S.7 Life cycle inventories

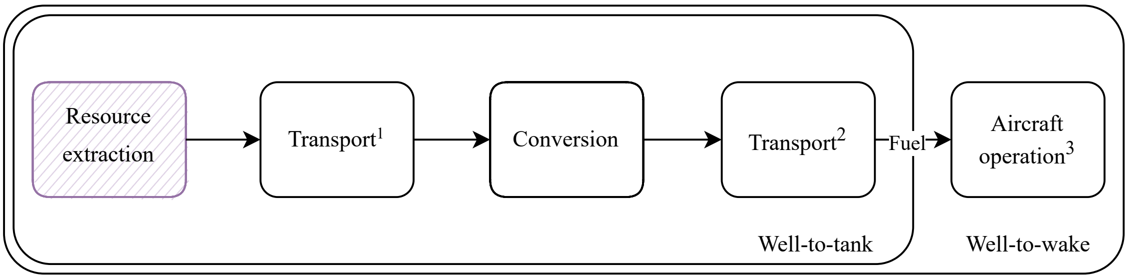

Figure S12: System stages considered in this well-to-wake study
[1]: Applicable for natural gas and crude oil
[2]: Not applicable for decentralised production of LH2 with PEM-electrolysis;
[3]: Modelled with the AviTeam

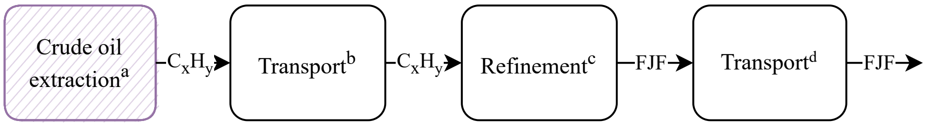

Figure S13: Fossil jet fuel (FJF) production
[a]: Supplementary Tbl. S5 - S8;
[b]: Supplementary Tbl. S9;
[c]: Supplementary Tbl. S10;
[d]: ecoinvent 3.8 (cutoff) process "transport, freight, lorry 16−32 metric ton, EURO5 [RER]" (Wernet et al., 2016).

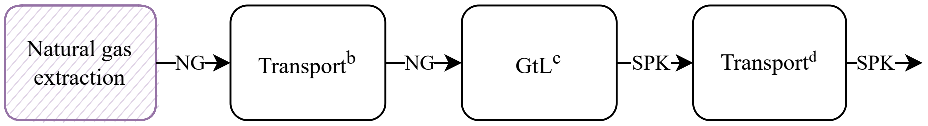

Figure S14: Synthesised paraffinic kerosene production in gas-to-liquid pathway
[a]: Supplementary Tbl. S5 − S11;
[b]: Supplementary Tbl. S12;
[c]: van der Giesen et al. (2014);
[d]: Supplementary Tbl. S16

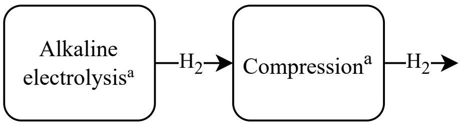

Figure S15: H2 production from alkaline electrolysis

[a]: van der Giesen et al. (2014)

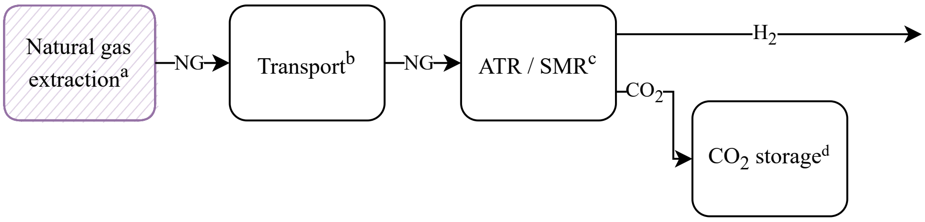

Figure S16: H2 production from NG with ATR or SMR

[a]: Supplementary Tbl. S5 − S11;

[b]: Supplementary Tbl. S12;

[c]: Antonini et al. (2020). ATR and SMR: 90% CCS with MDEA absorbent. H_2 output at 25 bar;

[d]: Antonini et al. (2020);

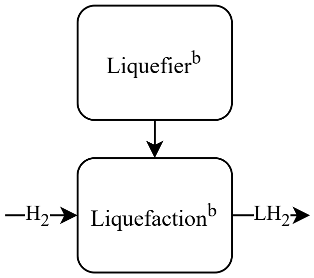

Figure S17: LH2 production from H2, decentralised

[b]: Akhtar et al. (2021);
[c]: Losses based on Teichmann et al. (2012)

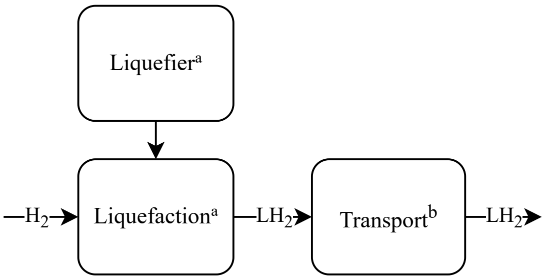

Figure S18: LH2 production, centralised

[a]: Akhtar et al. (2021);
[b]: Supplementary Tbl. S16;
[c]: Losses based on Teichmann et al. (2012).

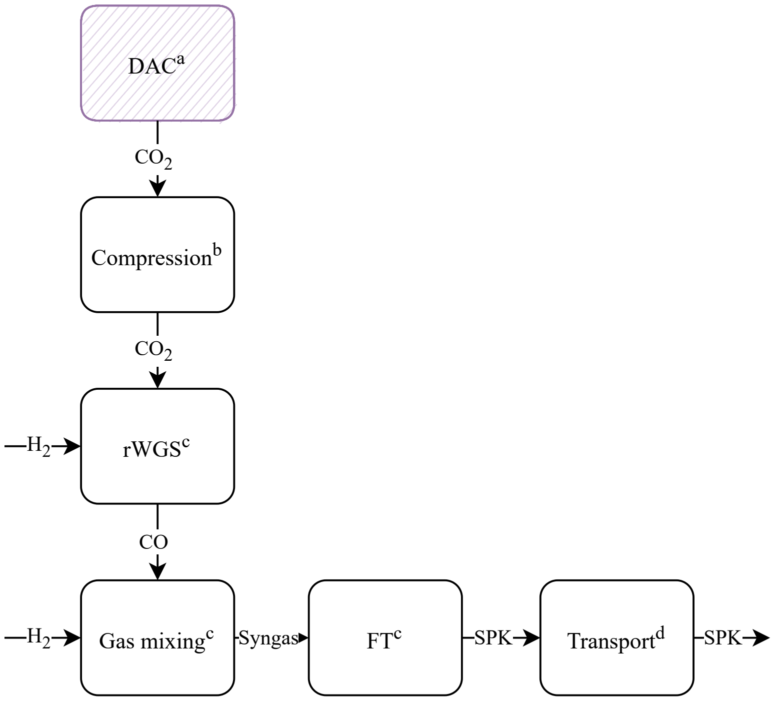

Figure S19: Synthesised paraffinic kerosene production from H2 and DAC

[a]: DAC with amine-based (Supplementary Tbl. S13) or calcium carbonate sorbent (Keith et al., 2018);
[b]: Supplementary Tbl. S15;
[c]: van der Giesen et al. (2014);
[d]: Supplementary Tbl. S16

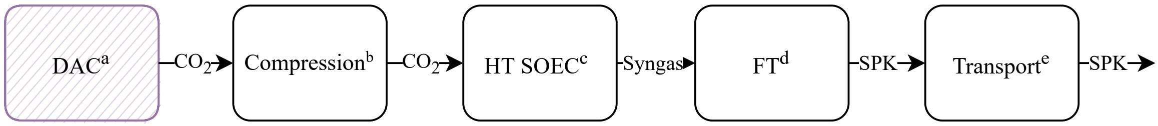

Figure S20: Synthesised paraffinic kerosene production with solid-oxide electrolysis

[a]: DAC with amine-based sorbent (Supplementary Tbl. S13);
[b]: Supplementary Tbl. S15;
[c]: (Schreiber et al., 2020)
[d]: (van der Giesen et al., 2014)
[e]: Supplementary Tbl. S16

Table S5: LCI of crude oil and natural gas extraction, Edvard Grieg platform.
Emissions from Norwegian Environment Agency (2023). Flows from ecoinvent 3.8 (cutoff) ”petroleum and gas production, off-shore [Norway]” (Wernet et al., 2016) marked with ”...”.

	Location	Amount	Unit
Outputs			
Crude oil or natural gas, Edvard Grieg	NO	1	MJ
Inputs			
...			
Biosphere			
Carbon dioxide, fossil		8.2282e-04	kg
Methane, fossil		4.4912e-07	kg
Nitrogen oxides		7.8529e-07	kg
NMVOC, non-methane volatile organic compounds		2.8439e-07	kg
Sulfur oxides		9.9304e-09	kg
Acenaphthene		8.1670e-15	kg
Acenaphthylene		2.4338e-15	kg
Anthracene		4.8231e-14	kg
Arsenic, ion		2.9808e-13	kg
Barium		1.4012e-10	kg
Benz(a)anthracene		1.1941e-15	kg
Benzene		5.5256e-11	kg
Benzene, ethyl-		2.1590e-12	kg
Benzo(a)pyrene		2.7800e-16	kg
Benzo(k)fluoranthene		1.4731e-15	kg
Benzo(ghi)perylene		7.3508e-16	kg
Benzo(k)fluoranthene		7.1232e-17	kg
Cadmium, ion		7.8553e-16	kg
Chromium, ion		1.5845e-14	kg
Chrysene		4.5371e-15	kg
Copper, ion		1.4831e-14	kg
Dibenz(a,h)anthracene		2.8295e-16	kg
Benzo(k)fluoranthene		8.0928e-16	kg
Fluorene		7.7531e-14	kg
Formic acid		1.2221e-11	kg
Hydrocarbons, aromatic		1.4538e-13	kg
Indeno(1,2,3-cd)pyrene		4.3400e-14	kg
Iron, ion		5.5433e-11	kg

Lead	1.2297e-15	kg
Mercury	1.0665e-15	kg
Nickel, ion	1.3000e-13	kg
Oils, unspecified	2.5186e-10	kg
PAH, polycyclic aromatic hydrocarbons	2.5212e-12	kg
PAH, polycyclic aromatic hydrocarbons	4.5222e-14	kg
PAH, polycyclic aromatic hydrocarbons	4.6767e-14	kg
Phenanthrene	1.5240e-13	kg
Phenol	4.2759e-11	kg
Indeno(1,2,3-cd)pyrene	3.0788e-15	kg
Toluene	4.8451e-11	kg
Xylene	2.0801e-11	kg
Zinc, ion	1.3570e-13	kg
Oil, crude, in ground	2.3333e-02	kg

Table S6: LCI of crude oil and natural gas extraction, Oseberg platform.
Emissions from Norwegian Environment Agency (2023). Flows from ecoinvent 3.8 (cutoff) "petroleum and gas production, off-shore [Norway]" (Wernet et al., 2016) marked with "...".

	Location	Amount	Unit
Outputs			
Crude oil or natural gas, Oseberg	NO	1	MJ
Inputs			
...			
Biosphere			
Carbon dioxide, fossil		1.1127e-03	kg
Methane, fossil		5.4018e-07	kg
Nitrogen oxides		4.4885e-06	kg
NMVOC, non-methane volatile organic compounds		5.5736e-07	kg
Sulfur oxides		1.5167e-08	kg
Acenaphthene		2.2680e-12	kg
Acenaphthylene		2.7938e-12	kg
Anthracene		1.4684e-11	kg
Arsenic, ion		1.1442e-12	kg
Barium		7.7996e-08	kg
Benz(a)anthracene		3.8086e-13	kg
Benzene		1.0378e-08	kg
Benzene, ethyl-		2.9520e-10	kg
Benzo(a)pyrene		1.3686e-13	kg
Benzo(k)fluoranthene		4.4876e-13	kg
Benzo(ghi)perylene		2.0129e-13	kg
Benzo(k)fluoranthene		1.5478e-13	kg
Cadmium, ion		6.0328e-14	kg
Chromium, ion		2.0703e-12	kg
Chrysene		2.1058e-12	kg
Copper, ion		2.1804e-13	kg
Dibenz(a,h)anthracene		6.8536e-14	kg
Benzo(k)fluoranthene		9.9890e-13	kg
Fluorene		2.1734e-11	kg
Formic acid		1.6411e-09	kg
Hydrocarbons, aromatic		3.8765e-11	kg
Indeno(1,2,3-cd)pyrene		6.7487e-14	kg

Iron, ion	8.9742e-09	kg
Lead	2.1749e-13	kg
Mercury	1.8052e-14	kg
Nickel, ion	2.1462e-12	kg
Oils, unspecified	3.6218e-08	kg
PAH, polycyclic aromatic hydrocarbons	6.6088e-10	kg
PAH, polycyclic aromatic hydrocarbons	8.0788e-13	kg
PAH, polycyclic aromatic hydrocarbons	2.0081e-12	kg
Phenanthrene	3.5362e-11	kg
Phenol	1.9626e-08	kg
Indeno(1,2,3-cd)pyrene	8.4039e-13	kg
Toluene	5.6426e-09	kg
Xylene	2.0612e-09	kg
Zinc, ion	2.3648e-11	kg
Oil, crude, in ground	2.3333e-02	kg

Table S7: LCI of crude oil and natural gas extraction, Statfjord platform.
Emissions from Norwegian Environment Agency (2023). Flows from ecoinvent 3.8 (cutoff) "petroleum and gas production, off-shore [Norway]" (Wernet et al., 2016) marked with "...".

	Location	Amount	Unit
Outputs			
Crude oil or natural gas, Statfjord	NO	1	MJ
Inputs			
...			
Biosphere			
Carbon dioxide, fossil		6.2094e-03	kg
Methane, fossil		5.9364e-06	kg
Nitrogen oxides		1.9894e-05	kg
NMVOC, non-methane volatile organic compounds		3.3632e-05	kg
Sulfur oxides		6.0487e-08	kg
Acenaphthene		2.0358e-10	kg
Acenaphthylene		1.9473e-10	kg
Anthracene		9.3867e-10	kg
Arsenic, ion		2.5278e-11	kg
Barium		1.9828e-06	kg
Benz(a)anthracene		1.8876e-11	kg
Benzene		1.5546e-06	kg
Benzene, ethyl-		6.5593e-08	kg
Benzo(a)pyrene		6.4992e-12	kg
Benzo(k)fluoranthene		2.6841e-11	kg
Benzo(ghi)perylene		8.0315e-12	kg
Benzo(k)fluoranthene		8.4882e-12	kg
Cadmium, ion		2.5909e-12	kg
Chromium, ion		1.2076e-10	kg
Chrysene		1.1833e-10	kg
Copper, ion		1.8758e-11	kg
Dibenz(a,h)anthracene		2.7538e-12	kg
Benzo(k)fluoranthene		5.7997e-11	kg
Fluorene		1.7849e-09	kg
Formic acid		6.0817e-07	kg
Hydrocarbons, aromatic		2.6002e-09	kg
Indeno(1,2,3-cd)pyrene		2.5001e-12	kg
Iron, ion		5.3375e-07	kg

Lead	5.3691e-12	kg
Mercury	2.0028e-12	kg
Nickel, ion	4.2877e-11	kg
Oils, unspecified	2.8754e-06	kg
PAH, polycyclic aromatic hydrocarbons	7.0902e-08	kg
PAH, polycyclic aromatic hydrocarbons	4.4328e-11	kg
PAH, polycyclic aromatic hydrocarbons	1.1036e-10	kg
Phenanthrene	2.4196e-09	kg
Phenol	7.4811e-07	kg
Indeno(1,2,3-cd)pyrene	5.3125e-11	kg
Toluene	1.0458e-06	kg
Xylene	3.4059e-07	kg
Zinc, ion	1.5750e-09	kg
Oil, crude, in ground	2.3333e-02	kg

Table S8: LCI of crude oil and natural gas extraction, Troll platform.
Emissions from Norwegian Environment Agency (2023). Flows from ecoinvent 3.8 (cutoff) "petroleum and gas production, off-shore [Norway]" (Wernet et al., 2016) marked with "...".

	Location	Amount	Unit
Outputs			
Crude oil or natural gas, Troll	NO	1	MJ
Inputs			
...			
Biosphere			
Carbon dioxide, fossil		3.9625e-04	kg
Methane, fossil		3.3075e-07	kg
Nitrogen oxides		2.2847e-06	kg
NMVOC, non-methane volatile organic compounds		3.3600e-07	kg
Sulfur oxides		1.8749e-08	kg
Acenaphthene		1.6573e-11	kg
Acenaphthylene		1.2016e-11	kg
Anthracene		1.5875e-11	kg
Arsenic, ion		8.1820e-13	kg
Barium		1.8014e-06	kg
Benz(a)anthracene		1.1293e-12	kg
Benzene		1.1812e-08	kg
Benzene, ethyl-		4.0776e-09	kg
Benzo(a)pyrene		4.1703e-13	kg
Benzo(k)fluoranthene		1.7741e-12	kg
Benzo(ghi)perylene		3.2659e-13	kg
Benzo(k)fluoranthene		4.4274e-13	kg
Cadmium, ion		2.4019e-13	kg
Chromium, ion		3.3405e-12	kg
Chrysene		6.9306e-12	kg
Copper, ion		1.9925e-12	kg
Dibenz(a,h)anthracene		1.1808e-13	kg
Benzo(k)fluoranthene		5.5759e-12	kg
Fluorene		9.2694e-11	kg
Formic acid		1.1799e-08	kg
Hydrocarbons, aromatic		1.4598e-10	kg
Indeno(1,2,3-cd)pyrene		1.4357e-13	kg
Iron, ion		9.5551e-08	kg

Lead	2.2507e-13	kg
Mercury	1.0376e-13	kg
Nickel, ion	5.5522e-12	kg
Oils, unspecified	1.2707e-07	kg
PAH, polycyclic aromatic hydrocarbons	3.4503e-09	kg
PAH, polycyclic aromatic hydrocarbons	2.7774e-12	kg
PAH, polycyclic aromatic hydrocarbons	8.6799e-12	kg
Phenanthrene	9.5907e-11	kg
Phenol	7.3257e-09	kg
Indeno(1,2,3-cd)pyrene	3.6379e-12	kg
Toluene	1.2474e-08	kg
Xylene	1.7298e-08	kg
Zinc, ion	7.2140e-12	kg

Table S9: LCI of crude oil transport to shore.
Distances calculated with data from the Norwegian Petroleum Directorate (Norwegian Petroleum Directorate, 2023).

	Location	Amount	Unit
Outputs			
Crude oil, at shore	NO	1	MJ
Inputs			
Pipeline construction, petroleum	RER	4.3387e-11	km
Market for transport, freight, sea, tanker for petroleum	GLO	1.8296e-01	ton km
Crude oil extraction, Edvard Grieg	NO	4.0884e-01	MJ
Crude oil extraction, Oseberg	NO	2.3245e-01	MJ
Crude oil extraction, Statfjord	NO	5.2570e-02	MJ
Crude oil extraction, Troll	NO	3.8972e-01	MJ

Table S11: LCI of natural gas extraction, off-shore, Kristin platform.

Emissions from Norwegian Environment Agency (2023). Flows from ecoinvent 3.8 (cutoff) "petroleum and gas production, off-shore [Norway]" (Wernet et al., 2016) marked with "...".

	Location	Amount	Unit
Outputs			
Natural gas, off-shore, Kristin	NO	1	m^3
Inputs			
...			
Biosphere			
Carbon dioxide, fossil		6.5972e-02	kg
Methane, fossil		7.0182e-05	kg
Nitrogen oxides		5.3214e-05	kg
NMVOC, non-methane volatile organic compounds		2.5953e-05	kg
Sulfur oxides		7.4333e-07	kg
Acenaphthene		3.4415e-09	kg
Acenaphthylene		7.8144e-09	kg
Anthracene		1.0536e-09	kg
Arsenic, ion		4.1032e-10	kg
Barium		2.7456e-04	kg
Benz(a)anthracene		1.1675e-11	kg
Benzene		4.0626e-06	kg
Benzene, ethyl-		1.5751e-07	kg
Benzo(a)pyrene		1.2182e-11	kg
Benzo(k)fluoranthene		2.8255e-11	kg
Benzo(ghi)perylene		1.2398e-11	kg
Benzo(k)fluoranthene		1.0389e-11	kg
Cadmium, ion		2.5620e-11	kg
Chromium, ion		3.8170e-10	kg
Chrysene		8.9446e-11	kg
Copper, ion		1.0177e-10	kg
Dibenz(a,h)anthracene		7.0552e-12	kg
Benzo(k)fluoranthene		3.6480e-11	kg
Fluorene		4.1874e-09	kg
Formic acid		2.8511e-06	kg
Hydrocarbons, aromatic		1.5776e-08	kg
Indeno(1,2,3-cd)pyrene		1.2703e-11	kg

Iron, ion	4.5334e-06	kg
Lead	1.8054e-09	kg
Mercury	1.2651e-10	kg
Nickel, ion	8.4016e-10	kg
Oils, unspecified	2.9174e-06	kg
PAH, polycyclic aromatic hydrocarbons	9.2933e-08	kg
PAH, polycyclic aromatic hydrocarbons	6.3530e-11	kg
PAH, polycyclic aromatic hydrocarbons	1.1241e-10	kg
Phenanthrene	1.8363e-09	kg
Phenol	5.1208e-06	kg
Indeno(1,2,3-cd)pyrene	4.6547e-11	kg
Toluene	2.3559e-06	kg
Xylene	6.5435e-07	kg
Zinc, ion	1.4731e-08	kg

Table S10: LCI of fossil jet fuel (FJF) production.
Emissions from Norwegian Environment Agency (2023). Flows from ecoinvent 3.8 (cutoff) ”kerosene production, petroleum refinery operation” (Wernet et al., 2016) marked with ”...”.

	Location	Amount	Unit
Outputs			
Fossil jet fuel	NO	1	MJ
Inputs			
...			
Crude oil, at shore	NO	1	MJ
Biosphere			
Chrysene		9.5040e-13	kg
PAH, polycyclic aromatic hydrocarbons		5.3881e-12	kg
PAH, polycyclic aromatic hydrocarbons		1.2604e-12	kg
Arsenic, ion		3.2114e-11	kg
Benz(a)anthracene		9.2994e-14	kg
Benzo(k)fluoranthene		5.4929e-13	kg
Benzo(a)pyrene		5.9454e-13	kg
Benzo(ghi)perylene		3.3416e-13	kg
Lead		1.4569e-11	kg
Cyanide		1.4673e-09	kg
Phenol		1.1277e-10	kg
Phosphorus		3.3788e-08	kg
Indeno(1,2,3-cd)pyrene		1.1655e-13	kg
Cadmium, ion		2.8766e-13	kg
Copper, ion		6.5778e-11	kg
Cobalt		2.0273e-11	kg
Chromium, ion		1.9411e-10	kg
Mercury		1.6782e-12	kg
Nickel, ion		1.8878e-10	kg
Nitrogen		1.5084e-07	kg
Oils, unspecified		8.9000e-09	kg
Indeno(1,2,3-cd)pyrene		7.0428e-13	kg
Zinc, ion		2.6485e-10	kg
Suspended solids, unspecified		1.1277e-07	kg
Vanadium, ion		3.3100e-10	kg
Cyanide		1.0986e-09	kg
Carbon dioxide, fossil		4.6774e-03	kg
Aluminium		3.8402e-09	kg
Arsenic		1.2709e-12	kg
Benzene		2.5350e-10	kg
Lead		1.1519e-11	kg
NMVOC, non-methane volatile organic compounds		8.2376e-06	kg
Copper		1.5406e-10	kg
Cobalt		2.4798e-13	kg
Mercury		4.3459e-12	kg
Manganese		2.5170e-10	kg
Methane, fossil		3.6203e-06	kg
Nitrogen oxides		3.4317e-06	kg
Particulates, > 10 um		2.6343e-07	kg
Zinc		8.0459e-10	kg
Sulfur dioxide		9.2472e-07	kg

Table S12: LCI of gas transport to shore

Outputs	Location	Amount	Unit
Natural gas, at shore	NO	1	m^3
**Inputs**			
Transport, pipeline, off-shore, long distance, natural gas	NO	2.3943e-01	ton km
Natural gas, off-shore, Edvard Grieg	NO	2.0394e-02	m^3
Natural gas, off-shore, Kristin	NO	7.8512e-02	m^3
Natural gas, off-shore, Oseberg	NO	2.2686e-01	m^3
Natural gas, off-shore, Statfjord	NO	3.6838e-02	m^3
Natural gas, off-shore, Troll	NO	6.3739e-01	m^3

Table S13: LCI of CO_2 production via direct air capture (DAC) with amine-based sorbent.

Based on Schreiber et al. (2020) and Deutz et al. (2021).

Outputs	Location	Amount	Unit
Carbon dioxide, 1 bar	NO	1	kg
**Inputs**			
Direct air capture unit[a]	NO	9.3000e-08[a]	unit
Electricity production, wind, <1MW turbine, onshore	NO	2.68[b]	kWh
Polyethyleneimine[b]	RoW	3.7500e-03[b]	kg
**Biosphere**			
Carbon dioxide, fossil		−1	kg

[a]:(Schreiber et al., 2020)

[b]:(Deutz et al., 2021)

Table S14: LCI of direct air capture with calcium carbonate adsorbent.
Based on Keith et al. (2018) and own modelling. As a simplification and to use electricity only, heat inputs are replaced by electricity in a ratio of 1:1. Biosphere flow updated to 1 kg CO_2 captured.

Outputs	Location	Amount	Unit
Carbon dioxide, 1 bar	NO	1	kg
**Inputs**			
Direct air capture unit[a]	NO	9.3000e-08[a]	unit
Market for calcium carbonate, precipitated	RER	2.0470e-02	kg
Market for oxygen, liquid	RER	3.3670e-01	kg
Electricity production, wind, <1MW turbine, onshore	NO	1.549	kWh
**Biosphere**			
Carbon dioxide, fossil		-1	kg

[a] (Schreiber et al., 2020)

Table S15: LCI of CO_2 compression from 1 bar to 25 bar.

Outputs	Location	Amount	Unit
Carbon dioxide, 25 bar	NO	1<br>0	kg
**Inputs**			
Carbon dioxide, 1 bar	NO	1	kg
Electricity production, wind, <1MW turbine, onshore[a]	NO	6.9491e-02	kWh
Compressor construction [b]	RER	2.9464e-06[b]	unit

[a] Compression energy derived from idealised isothermal compression work of gases (W)
$W = m * R * T * ln(\frac{p_{out}}{p_{in}})$(Brown, 2005).
[b] (van der Giesen et al., 2014)

Table S16: LCI of transport, freight, lorry 16−32 metric ton, hydrogen, liquefied.
Flow and quantities marked with ... from ecoinvent 3.8 (cutoff) process "transport, freight, lorry 16−32 metric ton, EURO5 [RER]" (Wernet et al., 2016).

Outputs	Location	Amount	Unit
Transport, freight, lorry 16−32 metric ton, hydrogen, liquefied	NO	1	ton km
**Inputs**			
Hydrogen from ATR, liquefied[a]	NO	...	MJ
...			
**Biosphere**			
Nitrogen oxides		...	kg

[a]: Modelled as in Supplementary Fig. S16

References

Agarwal, A., Speth, R. L., Fritz, T. M., Jacob, S. D., Rindlisbacher, T., Iovinelli, R., Owen, B., Miake-Lye, R. C., Sabnis, J. S. & Barrett, S. R. (2019). SCOPE11 Method for Estimating Aircraft Black Carbon Mass and Particle Number Emissions. *Environmental Science and Technology*, *53*(3), 1364–1373. https://doi.org/10.1021/acs.est.8b04060

Akhtar, M. S., Dickson, R. & Jay Liu, J. (2021). Life Cycle Assessment of Inland Green Hydrogen Supply Chain Networks with Current Challenges and Future Prospects. *Sustainable Chemical Engineering*, (9), 17152–17163. https://doi.org/10.1021/acssuschemeng.1c06769

Antonini, C., Treyer, K., Streb, A., van der Spek, M., Bauer, C. & Mazzotti, M. (2020). Hydrogen production from natural gas and biomethane with carbon capture and storage – A techno-environmental analysis. *Sustainable Energy & Fuels*, *4*(6), 2967–2986. https://doi.org/10.1039/D0SE00222D

Baldino, C., O'Malley, J., Searle, S., Zhou, Y. & Christensen, A. (2020). Hydrogen for heating? Decarbonization options for households in the United Kingdom in 2050. Retrieved 16/06/2023, from https://theicct.org/sites/default/files/publications/Hydrogen-heating-UK-dec2020.pdf

Ballal, V., Cavalett, O., Cherubini, F. & Watanabe, M. D. B. (2023). Climate change impacts of e-fuels for aviation in Europe under present-day conditions and future policy scenarios. *Fuel*, *338*, 127316. https://doi.org/10.1016/j.fuel.2022.127316

Bareiß, K., de La Rua, C., Möckl, M. & Hamacher, T. (2019). Life cycle assessment of hydrogen from proton exchange membrane water electrolysis in future energy systems. *Applied Energy*, *237*, 862–872. https://doi.org/10.1016/j.apenergy.2019.01.001

Bengtsson, S., Andersson, K. & Fridell, E. (2011). A comparative life cycle assessment of marine fuels: liquefied natural gas and three other fossil fuels. *Proceedings of the Institution of Mechanical Engineers, Part M: Journal of Engineering for the Maritime Environment*, *225*(2), 97–110. https://doi.org/10.1177/1475090211402136

Brown, R. N. (2005). *Compressors: Selection and sizing / Royce N. Brown* (3rd ed.). Elsevier.

Cain, J., DeWitt, M. J., Blunck, D., Corporan, E., Striebich, R., Anneken, D., Klingshirn, C., Roquemore, W. M. & Vander Wal, R. (2013). Characterization of Gaseous and Particulate Emissions From a Turboshaft Engine Burning Conventional, Alternative, and Surrogate Fuels. *Energy & Fuels*, *27*(4), 2290–2302. https://doi.org/10.1021/ef400009c

Cetinkaya, E., Dincer, I. & Naterer, G. F. (2012). Life cycle assessment of various hydrogen production methods. *International Journal of Hydrogen Energy*, *37*(3), 2071–2080. https://doi.org/10.1016/j.ijhydene.2011.10.064

Corporan, E., DeWitt, M. J., Belovich, V., Pawlik, R., Lynch, A. C., Gord, J. R. & Meyer, T. R. (2007). Emissions Characteristics of a Turbine Engine and Research Combustor Burning a Fischer−Tropsch Jet Fuel. *Energy & Fuels*, *21*(5), 2615–2626. https://doi.org/10.1021/ef070015j

Corporan, E., DeWitt, M. J., Klingshirn, C. D., Striebich, R. & Cheng, M.-D. (2010). Emissions Characteristics of Military Helicopter Engines with JP-8 and Fischer-Tropsch Fuels. *Journal of Propulsion and Power*, *26*(2), 317–324. https://doi.org/10.2514/1.43928

Corporan, E., Edwards, T., Shafer, L., DeWitt, M. J., Klingshirn, C., Zabarnick, S., West, Z., Striebich, R., Graham, J. & Klein, J. (2011). Chemical, Thermal Stability, Seal Swell, and Emissions Studies of Alternative Jet Fuels. *Energy & Fuels*, *25*(3), 955–966. https://doi.org/10.1021/ef101520v

Cox, B., Jemiolo, W. & Mutel, C. (2018). Life cycle assessment of air transportation and the Swiss commercial air transport fleet. *Transportation Research Part D: Transport and Environment*, *58*, 1–13. https://doi.org/10.1016/j.trd.2017.10.017

Deutz, S. & Bardow, A. (2021). Life-cycle assessment of an industrial direct air capture process based on temperature–vacuum swing adsorption. *Nature Energy*, *6*(2), 203–213. https://doi.org/10.1038/s41560-020-00771-9

DeWitt, M. J., Corporan, E., Graham, J. & Minus, D. (2008). Effects of Aromatic Type and Concentration in Fischer−Tropsch Fuel on Emissions Production and Material Compatibility. *Energy & Fuels*, *22*(4), 2411–2418. https://doi.org/10.1021/ef8001179

Drozd, G. T., Miracolo, M. A., Presto, A. A., Lipsky, E. M., Riemer, D. D., Corporan, E. & Robinson, A. L. (2012). Particulate Matter and Organic Vapor Emissions from a Helicopter Engine Operating on Petroleum and Fischer–Tropsch Fuels. *Energy & Fuels*, *26*(8), 4756–4766. https://doi.org/10.1021/ef300651t

Elgowainy, A., Han, J., Wang, M., Carter, N., Stratton, R., Hileman, J., Malwitz, A. & Balasubramanian, S. (2012). Life-Cycle Analysis of Alternative Avaition Fuels in GREET. Retrieved 16/06/2023, from https://publications.anl.gov/anlpubs/2016/05/127787.pdf

Ewing, M., Israel, B., Jutt, T., Talebian, H. & Stepanik, L. (2020). Hydrogen on the path to net-zero emissions: Costs and climate benefits. Retrieved 16/06/2023, from https://www.pembina.org/reports/hydrogen-climate-primer-2020.pdf

Filippone, A. & Bojdo, N. (2018). Statistical model for gas turbine engines exhaust emissions. *Transportation Research Part D: Transport and Environment*, *59*(February), 451–463. https://doi.org/10.1016/j.trd.2018.01.019

Forman, G. S., Hahn, T. E. & Jensen, S. D. (2011). Greenhouse Gas Emission Evaluation of the GTL Pathway. *Environmental Science and Technology*, *45*(20), 9084–9092. https://doi.org/10.1021/es202101b

Harper, J., Durand, E., Bowen, P., Pugh, D., Johnson, M. & Crayford, A. (2022). Influence of alternative fuel properties and combustor operating conditions on the

nvPM and gaseous emissions produced by a small-scale RQL combustor. *Fuel*, *315*, 123045. https://doi.org/10.1016/j.fuel.2021.123045

Huijbregts, M. A. J., Steinmann, Z. J. N., Elshout, P. M. F., Stam, G., Verones, F., Vieira, M., Zijp, M., Hollander, A. & van Zelm, R. (2017). ReCiPe2016: a harmonised life cycle impact assessment method at midpoint and endpoint level. *The International Journal of Life Cycle Assessment*, *22*(2), 138–147. https://doi.org/10.1007/s11367-016-1246-y

Hydrogen Council. (2021). Hydrogen decarbonization pathways: A life-cycle assessment. Retrieved 16/06/2023, from https://hydrogencouncil.com/wp-content/uploads/2021/04/Hydrogen-Council-Report_Decarbonization-Pathways_Part-1-Lifecycle-Assessment.pdf

ICAO. (2015). ICAO Doc 9976: Flight Planning and Fuel Management Manual (1st ed.).

ICAO. (2023). ICAO Engine Emissions Databank (V29B).

Keith, D. W., Holmes, G., St. Angelo, D. & Heidel, K. (2018). A Process for Capturing CO2 from the Atmosphere. *Joule*, *2*(8), 1573–1594. https://doi.org/10.1016/j.joule.2018.05.006

Khandelwal, B., Cronly, J., Ahmed, I. S., Wijesinghe, C. J. & Lewis, C. (2019). The effect of alternative fuels on gaseous and particulate matter (PM) emission performance in an auxiliary power unit (APU). *The Aeronautical Journal*, *123*(1263), 617–634. https://doi.org/10.1017/aer.2019.16

Klenner, J., Muri, H. & Strømman, A. H. (2022). High-resolution modeling of aviation emissions in Norway. *Transportation Research Part D: Transport and Environment*, *109*, 103379. https://doi.org/10.1016/j.trd.2022.103379

Kolb, S., Müller, J., Luna-Jaspe, N. & Karl, J. (2022). Renewable hydrogen imports for the German energy transition – A comparative life cycle assessment. *Journal of Cleaner Production*, *373*, 133289. https://doi.org/10.1016/j.jclepro.2022.133289

Lee, J. J., Lukachko, S. P., Waitz, I. A. & Schäfer, A. W. (2001). Historical and Future Trends in Aircraft Performance, Cost, and Emissions. *Annual Review of Energy and the Environment*, *Vol. 26*, 167–200. https://doi.org/10.1146/annurev.energy.26.1.167

Li-Jones, X., Penko, P. F., Williams, S. & Moses, C. (2007). Gaseous and Particle Emissions in the Exhaust of a T700 Helicopter Engine. *Volume 2: Turbo Expo 2007*, 395–411. https://doi.org/10.1115/GT2007-27522

Longden, T., Beck, F. J., Jotzo, F., Andrews, R. & Prasad, M. (2022). 'Clean' hydrogen? – Comparing the emissions and costs of fossil fuel versus renewable electricity based hydrogen. *Applied Energy*, *306*, 118145. https://doi.org/10.1016/j.apenergy.2021.118145

Lund, M. T., Aamaas, B., Berntsen, T., Bock, L., Burkhardt, U., Fuglestvedt, J. S. & Shine, K. P. (2017). Emission metrics for quantifying regional climate impacts of aviation. *Earth System Dynamics*, *8*(3), 1–33. https://doi.org/10.5194/esd-2017-11

Matzen, M. J., Alhajji, M. & Demirel, Y. (2015). Technoeconomics and Sustainability of Renewable Methanol and Ammonia Productions Using Wind Power-based Hydrogen. *Journal of Advanced Chemical Engineering*, *5*(3). https://doi.org/10.4172/2090-4568.1000128

Micheli, M., Moore, D., Bach, V. & Finkbeiner, M. (2022). Life-Cycle Assessment of Power-to-Liquid Kerosene Produced from Renewable Electricity and CO2 from Direct Air Capture in Germany. *Sustainability*, *14*, 10658. https://doi.org/10.3390/su141710658

Moore, R. H., Shook, M., Beyersdorf, A., Corr, C., Herndon, S., Knighton, W. B., Miake-Lye, R., Thornhill, K. L., Winstead, E. L., Yu, Z., Ziemba, L. D. & Anderson, B. E. (2015). Influence of Jet Fuel Composition on Aircraft Engine Emissions: A Synthesis of Aerosol Emissions Data from the NASA APEX, AAFEX, and ACCESS Missions. *Energy & Fuels*, *29*(4), 2591–2600. https://doi.org/10.1021/ef502618w

Moore, R. H., Thornhill, K. L., Weinzierl, B., Sauer, D., D'Ascoli, E., Kim, J., Lichtenstern, M., Scheibe, M., Beaton, B., Beyersdorf, A. J., Barrick, J., Bulzan, D., Corr, C. A., Crosbie, E., Jurkat, T., Martin, R., Riddick, D., Shook, M., Slover, G., ... Anderson, B. E. (2017). Biofuel blending reduces particle emissions from aircraft engines at cruise conditions. *Nature*, *543*(7645), 411–415. https://doi.org/10.1038/nature21420

Myhre, G., Shindell, D., Bréon, F.-M., Collins, W., Fuglestvedt, J., Huang, J., Koch, D., Lamarque, J.-F., Lee, D. S., Mendoza, B., Nakajima, T., Robock, A., Stephens, G., Takemura, T. & Zhang, H. (2013). Anthropogenic and natural radiative forcing. In T. F. Stocker, D. Qin, G.-K. Plattner, M. Tignor, S. K. Allen, J. Doschung, A. Nauels, Y. Xia, V. Bex & P. M. Midgley (Eds.), *Climate Change 2013: The Physical Science Basis. Contribution of Working Group I to the Fifth Assessment Report of the Intergovernmental Panel on Climate Change* (pp. 659–740). Cambridge University Press. https://doi.org/10.1017/CBO9781107415324.018

Norwegian Environment Agency. (2023). Total emissions to air in Norway. Retrieved 18/05/2023, from https://www.norskeutslipp.no/en/Articles/About-Norske-Utslipp/

Norwegian Petroleum Directorate. (2023). Open data. Retrieved 30/05/2023, from https://www.npd.no/en/about-us/open-data/

Nuic, A., Poles, D. & Mouillet, V. (2010). BADA: An advanced aircraft performance model for present and future ATM systems. *International Journal of Adaptive Control and Signal Processing*, *24*(10), 850–866. https://doi.org/10.1002/acs.1176

Ocko, I. B. & Hamburg, S. P. (2022). Climate consequences of hydrogen emissions. *Atmospheric Chemistry and Physics*, *22*(14), 9349–9368. https://doi.org/10.5194/acp-22-9349-2022

Oni, A. O., Anaya, K., Giwa, T., Di Lullo, G. & Kumar, A. (2022). Comparative assessment of blue hydrogen from steam methane reforming, autothermal

reforming, and natural gas decomposition technologies for natural gas-producing regions. *Energy Conversion and Management*, *254*, 115245. https://doi.org/10.1016/j.enconman.2022.115245

Peck, J., Oluwole, O. O., Wong, H.-W. & Miake-Lye, R. C. (2013). An algorithm to estimate aircraft cruise black carbon emissions for use in developing a cruise emissions inventory. *Journal of the Air & Waste Management Association (1995)*, *63*(3), 367–375. https://doi.org/10.1080/10962247.2012.751467

Quadros, F. D. A., Snellen, M., Sun, J. & Dedoussi, I. C. (2022). Global Civil Aviation Emissions Estimates for 2017–2020 Using ADS-B Data. *Journal of Aircraft*, 1–11. https://doi.org/10.2514/1.C036763

Rooijers, F. & van Cappellen, L. (2018). Feasibility study into blue hydrogen: Technical, economic and sustainability analysis. Retrieved 16/06/2023, from https://cedelft.eu/wp-content/uploads/sites/2/2021/04/CE_Delft_9901_Feasibility_study_into_blue_hydrogen_DEF_bak.pdf

Schreiber, A., Peschel, A., Hentschel, B. & Zapp, P. (2020). Life Cycle Assessment of Power-to-Syngas: Comparing High Temperature Co-Electrolysis and Steam Methane Reforming. *Frontiers in Energy Research*, *8*. https://doi.org/10.3389/fenrg.2020.533850

Schripp, T., Anderson, B., Crosbie, E. C., Moore, R. H., Herrmann, F., Oßwald, P., Wahl, C., Kapernaum, M., Köhler, M., Le Clercq, P., Rauch, B., Eichler, P., Mikoviny, T. & Wisthaler, A. (2018). Impact of Alternative Jet Fuels on Engine Exhaust Composition During the 2015 ECLIF Ground-Based Measurements Campaign. *Environmental Science and Technology*, *52*(8), 4969–4978. https://doi.org/10.1021/acs.est.7b06244

Skone, T. J., Littlefield, J., Marriott, J., Cooney, G., Demetrion, L., Jamieson, M., Jones, C., Mutchek, M., Shih, C. Y., Schivley, G. & Krynock, M. (2016). Life Cycle Analysis of Natural Gas Extraction and Power Generation: DOE/NETL-2015/1714. Retrieved 30/05/2023, from https://www.netl.doe.gov/projects/files/LifeCycleAnalysisofNaturalGasExtractionandPowerGeneration_083016.pdf

Snijders, T. A., Melkert, J., Bogers, Paul. F., Bauldreay, Joanna. M., Wahl, C. & Kapernaum, M. G. (2011). Impact of fuel composition on emissions and performance of GTL kerosene blends in a Cessna Citation II. *12th International Conference on Stability, Handling and Use of Liquid Fuels.*

Snyder, C. A., Berton, J. J., Brown, G. V., Dolce, J. L., Dravid, M. V., Eichenberg, D. J., Freeh, J. E., Gallo, C. A., Jones, S. M., Kundu, K. P., Marek, C. J., Millis, M. G., Murthy, P. L., Roach, T., Smith, Timothy, Stefko, G., Sullivan, R. M., Tornabene, R. T., … Kascak, A. F. (2009). Propulsion Investigation for Zero and Near-Zero Emissions Aircraft.

Statistics Norway. (2023a). Data Table 08510: Air transport: Passengers between Norwegian airports (closed time series) 2009K1 - 2022K1. Retrieved 18/05/2023, from https://www.ssb.no/en/statbank/table/08510/

Statistics Norway. (2023b). Data Table 08511: Air transport: Aircraft movements and available seats between Norwegian airports 2009M01 - 2023M04. Retrieved 18/05/2023, from https://www.ssb.no/en/statbank/table/08510/

Teichmann, D., Arlt, W. & Wasserscheid, P. (2012). Liquid Organic Hydrogen Carriers as an efficient vector for the transport and storage of renewable energy. *International Journal of Hydrogen Energy*, *37*(23), 18118–18132. https://doi.org/10.1016/j.ijhydene.2012.08.066

Timko, M. T., Herndon, S. C., de La Rosa Blanco, E., Wood, E. C., Yu, Z., Miake-Lye, R. C., Knighton, W. B., Shafer, L., DeWitt, M. J. & Corporan, E. (2011). Combustion Products of Petroleum Jet Fuel, a Fischer–Tropsch Synthetic Fuel, and a Biomass Fatty Acid Methyl Ester Fuel for a Gas Turbine Engine. *Combustion Science and Technology*, *183*(10), 1039–1068. https://doi.org/10.1080/00102202.2011.581717

Undavalli, V., Hamilton, J., Ubogu, E., Ahmed, I. & Khandelwal, B. (2022). Impact of HEFA Fuel Properties on Gaseous Emissions and Smoke Number in a Gas Turbine Engine. *Volume 3B: Combustion, Fuels, and Emissions.* https://doi.org/10.1115/GT2022-82201

van der Giesen, C., Kleijn, R. & Kramer, G. J. (2014). Energy and climate impacts of producing synthetic hydrocarbon fuels from CO(2). *Environmental Science and Technology*, *48*(12), 7111–7121. https://doi.org/10.1021/es500191g

Verstraete, D., Hendrick, P., Pilidis, P. & Ramsden, K. (2010). Hydrogen fuel tanks for subsonic transport aircraft. *International Journal of Hydrogen Energy*, *35*(20), 11085–11098. https://doi.org/10.1016/j.ijhydene.2010.06.060

Warwick, N., Griffiths, P., Keeble, J., Archibald, A., Pyle, J. & Shine, K. P. (2022). Atmospheric implications of increased hydrogen use. Retrieved 23/05/2023, from https://assets.publishing.service.gov.uk/government/uploads/system/uploads/attachment_data/file/1067144/atmospheric-implications-of-increased-hydrogen-use.pdf

Wernet, G., Bauer, C., Steubing, B., Reinhard, J., Moreno-Ruiz, E. & Weidema, B. (2016). The ecoinvent database version 3 (part I): overview and methodology. *The International Journal of Life Cycle Assessment*, *21*(9), 1218–1230. https://doi.org/10.1007/s11367-016-1087-8

Williams, P. I., Allan, J. D., Lobo, P., Coe, H., Christie, S., Wilson, C., Hagen, D., Whitefield, P., Raper, D. & Rye, L. (2012). Impact of alternative fuels on emissions characteristics of a gas turbine engine - part 2: volatile and semivolatile particulate matter emissions. *Environmental Science and Technology*, *46*(19), 10812–10819. https://doi.org/10.1021/es301899s

Yugo, M., Gordillo, V., Shafiei, E. & Megaritis, A. (2021). A look into the life cycle assessment of passenger cars running on advanced fuels. *Proceedings of the SIA Powertrains & Power Electronics Conference, Paris, France*, 9–10.

Zhang, J., Ling, B., He, Y., Zhu, Y. & Wang, Z. (2022). Life cycle assessment of three types of hydrogen production methods using solar energy. *International Journal of Hydrogen Energy*, *47*(30), 14158–14168. https://doi.org/10.1016/j.ijhydene.2022.02.150

Zhao, G., Kraglund, M. R., Frandsen, H. L., Wulff, A. C., Jensen, S. H., Chen, M. & Graves, C. R. (2020). Life cycle assessment of H2O electrolysis technologies. *International Journal of Hydrogen Energy*, *45*(43), 23765–23781. https://doi.org/10.1016/j.ijhydene.2020.05.282
